# Subglacial geology and thermal conditions regulate methane emissions from Svalbard glaciers

**DOI:** 10.1038/s41467-026-77190-z

**Published:** 2026-09-08

**Authors:** Gabrielle E. Kleber, Erik Schytt Mannerfelt, Stefan Schloemer, Kim Senger, Leonard Magerl, Geert Hensgens, Ursula Enzenhofer, Jamie Hollander, Louise Steffensen Schmidt, Silje Waaler, Andrew J. Hodson

**Affiliations:** 1 Department of Geoscience, UiT the Arctic University of Norway, Tromsø, Norway; 2 Department of Geosciences, University of Oslo, Oslo, Norway; 3 Arctic Geology, University Centre in Svalbard (UNIS), Longyearbyen, Norway; 4 BGR – Federal Institute for Geosciences and Natural Resources, Hanover, Germany; 5 Department of Geography and Social Anthropology, Norwegian University of Science and Technology (NTNU), Trondheim, Norway; 6 Department of Civil Engineering and Environmental Sciences, Western Norway University of Applied Sciences, Sogndal, Norway

**Keywords:** Carbon cycle, Hydrology, Environmental chemistry, Cryospheric science

## Abstract

Melt rivers from valley glaciers and ice sheets release methane by transporting microbial or geologic methane from beneath glaciers, making it available for atmospheric emission. However, the mechanisms and magnitude of such methane release remain poorly understood due to limited studies. Here, we show how subglacial geology and glacier thermal conditions control methane supersaturation in meltwaters from 19 valley glaciers across central Svalbard. We find that all sampled meltwaters are supersaturated with methane, with peak concentrations up to 425-times atmospheric equilibrium. The carbon isotopic compositions of the methane and the presence of ethane and propane indicate that the methane is largely of thermogenic origin. Ground-penetrating radar surveys show that the extent of temperate ice at the glacier bed plays a critical role in facilitating methane acquisition, particularly in regions underlain by organic-rich, shale-dominated geological formations. This mechanistic insight is relevant for glaciated regions worldwide with organic-rich geology and suggests that accelerated glacier melt may amplify methane mobilization from beneath glaciers.

## Abstract (web summary)

The authors show that meltwaters from below Svalbard glaciers transport ancient, geologic methane. Methane mobilization is greatest where thawed glacier beds overlie organic-rich shale, suggesting that increased melt may initially enhance emissions.

## Introduction

Rapidly rising air temperatures in the Arctic^1^ are triggering cascading effects due to the temperature sensitivity of high-latitude environments. Glacier melt and permafrost thaw are initiating feedback loops that enhance natural methane emissions^2,3^—a powerful greenhouse gas—thereby amplifying the ongoing warming trend. The Intergovernmental Panel on Climate Change (IPCC) has identified significant uncertainty regarding the extent to which these Arctic emissions contribute to rising global atmospheric methane concentrations. Additionally, there is limited understanding of how these emissions may intensify with further warming^4^. This knowledge gap is largely attributed to the challenges of accessing remote Arctic regions and the resulting scarcity of direct measurements, leaving critical temperature-dependent mechanisms of methane release poorly understood.

The Arctic hosts a vast reservoir of carbon^5^, a large portion of which exists as methane stored in geologic natural gas deposits and gas hydrates. Glaciers can facilitate the migration of this methane to the subglacial environment through processes such as glacial erosion and geological faulting caused by the overburden pressure of glacier ice^6,7^. Additionally, as glaciers advance, they bury vegetation and soil, capturing subglacial reservoirs of organic carbon that can be microbially converted into methane^8^. Glaciers then act as a barrier, or cryospheric cap^2,9^, sequestering methane and inhibiting its release to the atmosphere. Furthermore, glaciers can create and maintain the high-pressure, low-temperature conditions required for methane to be stored as gas hydrates, where methane molecules are caged within an ice-like lattice of water molecules and can hold substantially more gas per unit volume^10^.

The temperature and hydrological conditions of the subglacial environment are governed by the glacier’s thermal regime, or the extent of cold (sub-freezing) versus temperate (warm, above the pressure melting point) ice. Cold-based glaciers have frozen beds and underlying permafrost that can penetrate deep into the bedrock, which limits subglacial hydraulic conductivity^11–14^. Conversely, temperate glaciers maintain thawed, hydrologically active beds^12–15^. Polythermal glaciers, which are widespread across much of the Arctic, contain a mix of temperate and cold ice at their beds, resulting in subglacial drainage systems that are more spatially variable compared to those of fully temperate glaciers^14^. Glacier retreat may enhance the release of methane trapped beneath a glacier by increasing meltwater inputs and stimulating hydrological activity at the bed, provided temperate ice is present. Additionally, glacier thinning and the associated reduction in subglacial pressure may destabilize gas hydrates^10^, thus facilitating the transport of methane via meltwater.

A limited number of studies across the Arctic and sub-Arctic have investigated the mobilization of subglacial methane by glacial meltwaters^16–21^. Most studies report that meltwaters are methane-supersaturated relative to atmospheric equilibrium, indicating that glacial rivers act as a net source of methane to the atmosphere. Svalbard, a Norwegian archipelago in the high Arctic with an abundance of polythermal glaciers, provides a diverse range of glacier bed conditions for investigating subglacial methane mobilization. One extensively studied glacial catchment, Vallåkrabreen, demonstrates that ancient thermogenic methane is flushed from the subglacial geology by glacial meltwaters^18^. Given that glaciers cover nearly 60% of the archipelago^22^ and methane-rich source rocks occupy almost half of Svalbard’s land surface area^23^, the methane emissions resulting from glacial meltwater flushing are expected to exceed those from permafrost thaw in the region^18,24^. While permafrost thaw on Svalbard can result in high methane emissions per unit area^25^, the organic-rich soils and wetlands that yield high rates of methane production are not expansive on the archipelago. Consequently, methane emissions from Svalbard’s extensive glacial catchments may be a more significant source of methane to the atmosphere^24^.

We undertook a comprehensive field study to determine whether high methane concentrations are widespread in glacial runoff across a range of glacial thermal and geological conditions. Our study focused on Svalbard, where the low elevation of glaciers makes them especially vulnerable to warming temperatures, placing them in a more advanced stage of deglaciation compared to many other glaciated regions^26^. We measured methane concentrations along transects of 19 glacial rivers across central Svalbard, covering a range of geological settings and land-terminating glacier sizes. Our hydro-chemical measurements were accompanied by ground-penetrating radar (GPR) surveys on a selection of glaciers to evaluate how glacial thermal regime and bedrock geology influence methane dynamics. Our findings classify the origins of the methane, identify key factors controlling methane supersaturation in glacial meltwaters, and provide a first-order estimate of the methane mobilized by glacial rivers across Svalbard.

## Results and discussion

### Pervasive methane supersaturation in glacial meltwaters

Glacial river samples were collected in transects along the length of each river, beginning as close as possible to the glacier terminus and extending downstream beyond the glacier end moraine or to the river’s outlet at the fjord. Methane concentrations at the glacier termini are used to infer the amount of methane transported from beneath the glacier. Sampling along the river transects provides insights into the downstream fate of the methane, including potential outgassing to the atmosphere and contributions from additional methane sources, such as glacier-fed groundwaters. The isotopic composition of the methane combined with the wetness ratio (C_1_/(C_2_ + C_3_)) indicates the likely origin of the methane, either thermogenic (geologic) or microbial. Changes in isotopic signatures along the rivers offer evidence of microbial processes that influence methane concentrations.

**Fig. 1:**
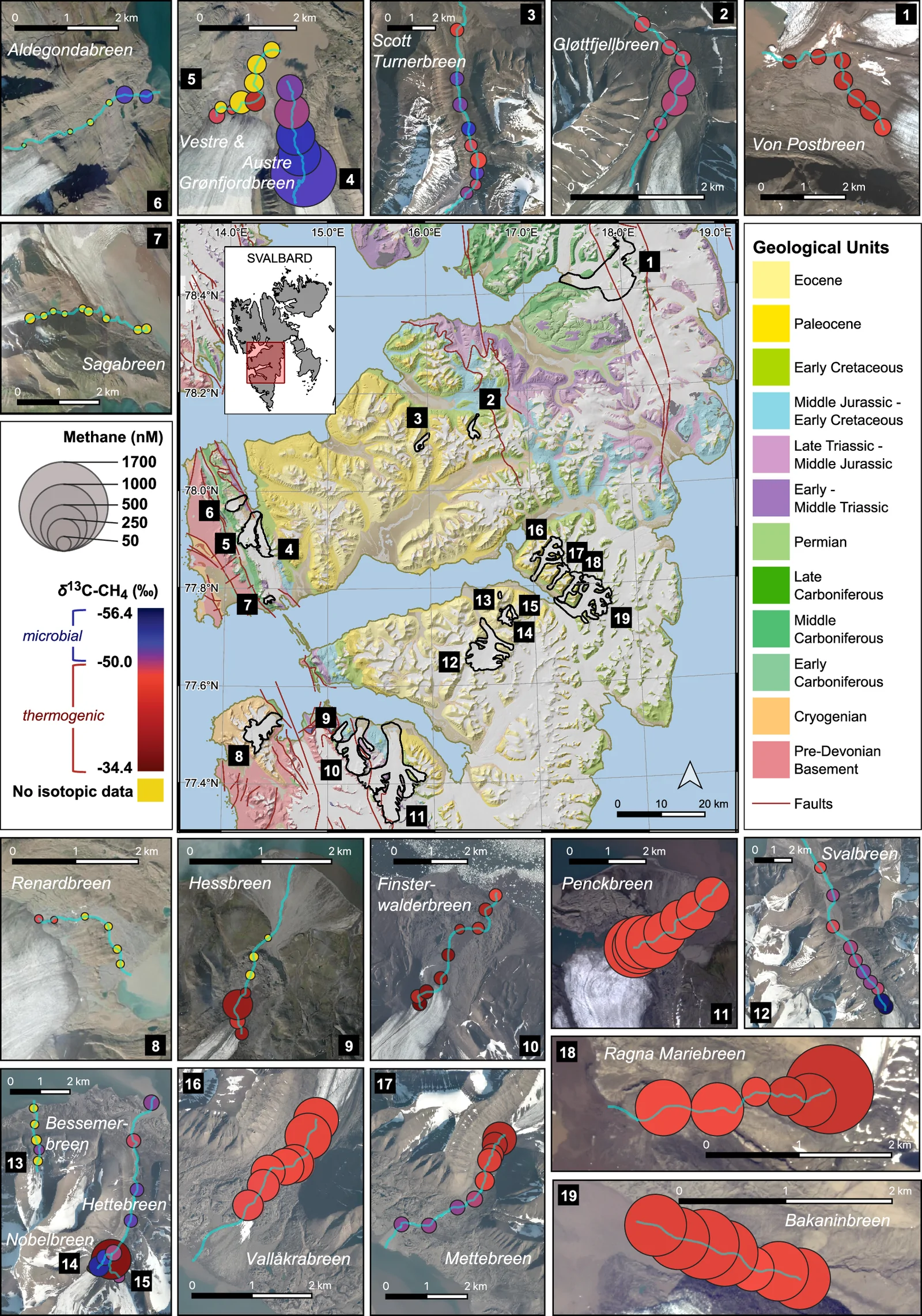
Overview of river sampling sites and corresponding methane concentrations. Geological map of central Svalbard (see inset map for location) with the surveyed glacial catchments outlined in black. Methane concentrations (nM) of river samples are displayed by bubble size over aerial or satellite imagery. The size scale applies equally to all transects. Bubble color denotes the carbon isotopic composition of the methane (*δ*^13^C-CH_4_, ‰), with blue indicating a typical microbial signature (<−50‰), red indicating a typical thermogenic signature (>−50‰), and yellow indicating that no isotopic data is available. River flowpaths are drawn in light blue. Aerial imagery © Norwegian Polar Institute, satellite imagery from Esri World Imagery (sources: Esri, Vantor) and Planet Labs (source: Copernicus Sentinel). The geology basemap is adapted from data sets available at https://geodata.npolar.no/ with permission under a Creative Commons license CC BY 4.0.

Our analysis of 148 river samples from 19 valley glaciers reveals widespread methane supersaturation in glacial meltwaters across Svalbard, with concentrations exceeding atmospheric equilibrium by up to 425-times. Methane concentrations measured as close as possible to the glacier termini range from 9 to 1700 nM (mean: 244 nM, median: 46 nM, *n* = 19), with stable carbon isotopic compositions of that methane ranging from −56.4 to −34.4‰ (mean: −46.6‰, median: −47.8‰, *n* = 15) (Fig.1). In glacial rivers, where water temperatures are near 0 °C, methane concentrations above ~4 nM are out of equilibrium with the atmosphere and methane begins to diffuse, or outgas, as the rivers flow downstream.

### Largely thermogenic methane is supplemented by instream methanogenesis

The carbon isotopic compositions (δ^13^C–CH_4_) of methane emerging with meltwaters at the glacier termini predominantly fall within the thermogenic range. Microbial methane, produced through the microbially-mediated breakdown of organic matter, typically exhibits δ^13^C values between −110 and −50‰^27^. In contrast, thermogenic methane, generated abiotically through the breakdown of organic matter at high temperatures and pressures, is less depleted in ^13^C, with δ^13^C values ranging from −50 to −20‰^27^. Among the 19 glacial rivers studied, only three exhibit isotopic signatures indicative of microbially produced methane (δ^13^C < −50‰) at the glacier terminus, suggesting that the methane transported by meltwaters is predominantly thermogenic in origin.

It is important to note that the isotopic composition of methane can be altered by microbial oxidation, or methanotrophy, a process which has been found to occur in the subglacial environments on Greenland^28,29^. Methanotrophy enriches the remaining methane in ^13^C, causing its isotopic signature to shift toward the thermogenic range. However, we suspect that the thermogenic signatures observed in our study are not primarily the result of subglacial methane oxidation, but instead reflect the abundant sources of thermogenic methane present in Svalbard’s organic-rich geology.

**Fig. 2:**
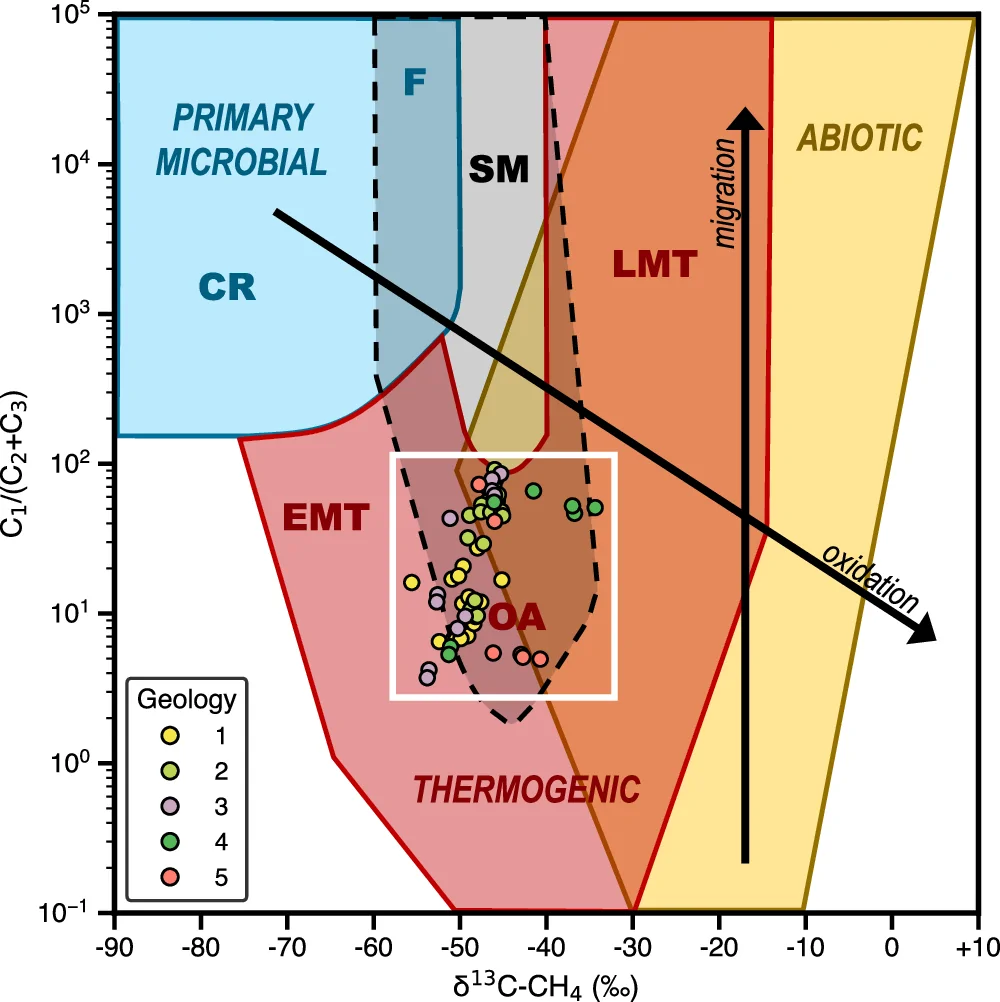
Wetness levels of river water plotted against stable carbon isotopic signatures of methane. δ^13^C–CH_4_ (‰) versus wetness (C_1_/(C_2_ + C_3_)) of river transect samples (circles, *n* = 61 from 12 of our rivers) plotted on top of a genetic diagram adapted from ref. ^37^. The color of the circle is representative of the dominant geology of the catchment: (1) Paleocene and Eocene, (2) Early Cretaceous, (3) Triassic-Middle Jurassic, (4) Carboniferous and Permian, (5) Pre-Devonian Basement. **CR**: CO_2_ reduction, **F**: methyl-type fermentation, **SM**: secondary microbial, **EMT**: early mature thermogenic gas, **OA**: oil-associated thermogenic gas, **LMT**: late mature thermogenic gas. Black arrows indicate the direction the wetness and/or isotopic compositions would shift due to methane oxidation or gas migration. The white box represents the region in which all our measured river samples are plotted.

The geology of Svalbard contains extensive hydrocarbon systems^30,31^ and numerous thick methane-rich source rocks, including coal seams and shale^32,33^. Natural seepages of this methane are found both onshore and offshore Svalbard^2,34–36^. Ethane and propane concentrations measured in our river samples yield wetness (C_1_/(C_2_ + C_3_)) levels ranging from 3.7 to 91.4 (*n* = 61). When coupled with the carbon isotopic signatures, these wetness levels indicate that the methane originates from oil-associated, early mature or late mature thermogenic gas^37^ (Fig. 2). These findings are consistent with previous geochemical observations of gaseous hydrocarbons liberated from rocks in the same geological settings on Svalbard, where gases were found to be largely of thermogenic origin and varying degrees of maturity^33^.

Our findings are in contrast to those from glacial rivers in Greenland, where crystalline bedrock dominates, and the methane in meltwaters is of microbial origin, produced subglacially through the breakdown of overridden vegetation^16,19,21,29^. While subglacial environments on Svalbard also presumably offer the conditions and substrate necessary for microbial methane production^38^, the abundance of thermogenic methane from organic-rich bedrock overwhelmingly dominates in most catchments. During glacial erosion, the grinding of organic-rich bedrock liberates thermogenic methane, which accumulates in the subglacial environment. Glacial grinding has been found to liberate methane at a greater rate than microbial methane is generated in the same subglacial sediments^39^.

For example, a study of the Vallåkrabreen glacier on Svalbard found that its meltwaters transport up to six times more methane per glacier area during the melt season than the meltwaters of Leverett Glacier in Greenland^18^. This difference arises because Leverett lies on crystalline bedrock, which is unsuitable for hydrocarbon storage, and its meltwaters contain primarily subglacially-produced microbial methane^16^. In contrast, Vallåkrabreen’s subglacial waters flush thermogenic methane from fractures in the underlying organic-rich shale, resulting in greater methane mobilization^18^. Additionally, hydraulic pressure during periods of high subglacial water flow may contribute to rock fracturing^40,41^, further enhancing methane release. These natural processes of erosion and fracturing are highly effective at mobilizing methane in subglacial environments and are likely common in glaciers overriding organic-rich rocks such as shale—environments that are widespread across many parts of the Arctic.

**Fig. 3:**
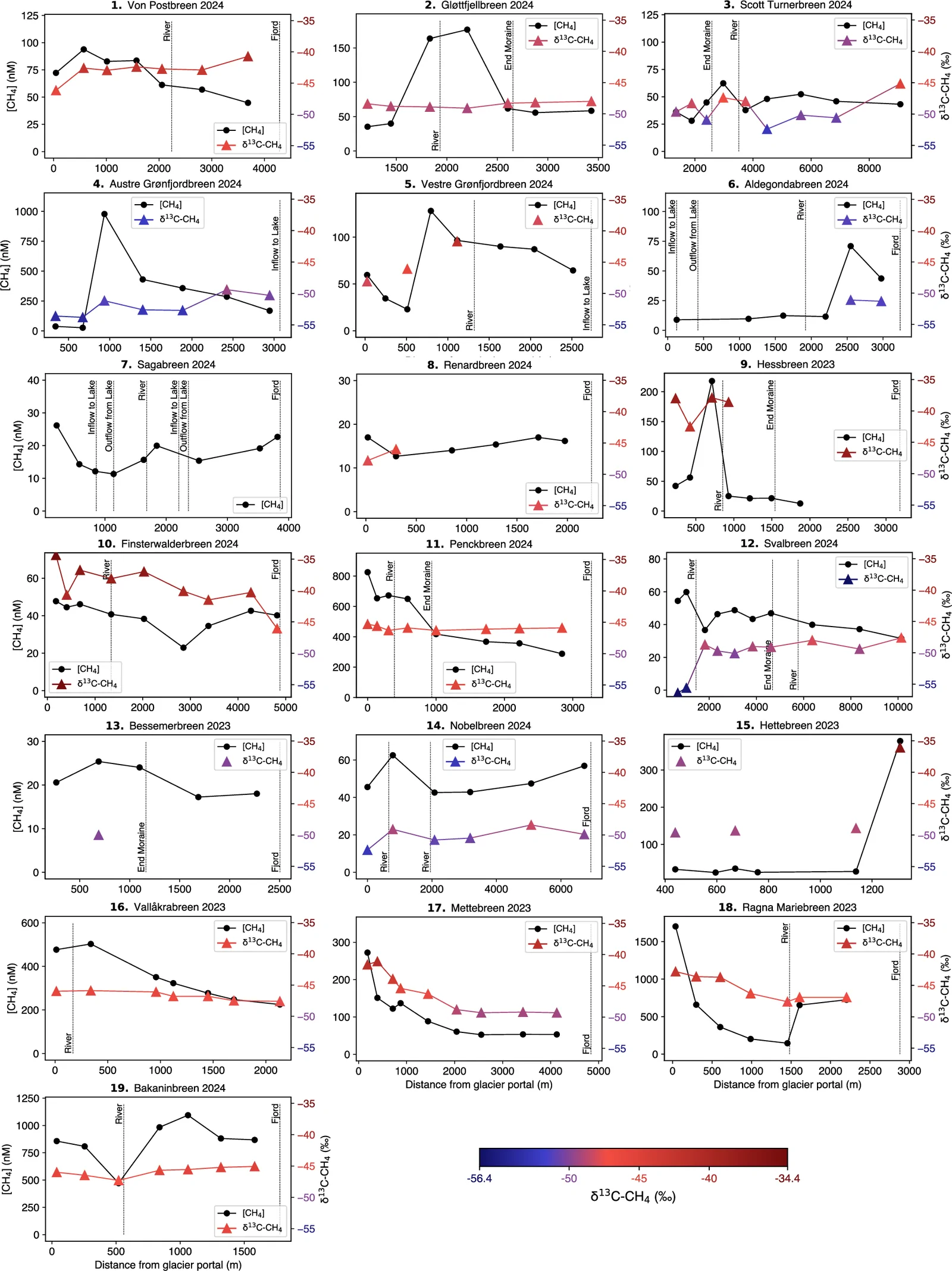
Transects of methane concentration and carbon isotopic composition. Methane concentrations ([CH_4_], nM) and the carbon isotopic compositions of methane (δ^13^C–CH_4_, ‰) are plotted in transects along the length of each river. Glacier catchments are numbered according to the labels in Fig. 1. The δ^13^C–CH_4_ is colored on a scale according to its value, with blue indicating the typical microbial range (<−50‰) and red indicating the thermogenic range (>−50‰). Each plot uses the same color scale denoted in the legend.

Our transects generally show a downstream decrease in methane concentrations along the rivers, likely due primarily to outgassing to the atmosphere. However, we also observe frequent additional recharge of methane along the river flowpaths. Abrupt increases in methane concentration downstream from the glacier terminus, such as those observed in the Gløttfjellebreen (2) and Vestre Grønfjordbreen (5) rivers (Fig. 3), are likely caused by discrete inputs of methane-rich groundwaters, which are commonly found in glacier forefields and can be from glacial or non-glacial sources^2,18,42–44^. Furthermore, isotopic changes along some rivers suggest that, while methane is being lost to the atmosphere through outgassing, it is also being replenished by the microbial production of methane (methanogenesis) in riverbed sediments. During the microbial breakdown of organic matter into methane, kinetic isotopic fractionation enriches the pool of methane in lighter carbon isotopes (^12^C)^27^. In contrast, isotopic fractionation during gas transfer from water to air is negligible, especially in turbulent conditions^45^. The isotopic enrichment in ^12^C is evident in some rivers, where downstream isotopic compositions become progressively more negative (e.g., Vallåkrabreen (16) and Mettebreen (17) rivers, Fig. 3), suggesting a small, continuous source of microbial methane. Additional evidence for instream methanogenesis comes from an incubation experiment with meltwater from Svalbreen (12), which yielded a > 40% increase in methane concentration after 30 h (Supplementary Fig. 1).

In contrast, we observe the opposite isotopic trend in some rivers, where the methane becomes progressively enriched in ^13^C, and thus the δ^13^C–CH_4_ values become less negative along the flowpath (e.g. in the second half of the Gløttfjellbreen (2) and Penckbreen (11) rivers, Fig. 3). This is suggestive of microbial methanotrophy, a process in which methane is consumed and oxidized into carbon dioxide under aerobic conditions. Like methanogenesis, methanotrophy is associated with kinetic isotopic fractionation, which enriches the residual methane in heavier carbon isotopes (^13^C)^27^. Methanotrophy acts as a methane sink and has the potential to moderate methane emissions in glacial environments^20,28,46,47^. We have evidence of active methanotrophy in groundwater streams feeding into the glacial rivers, supported by an incubation study (Supplementary Fig. 2) and previous isotopic analyses^18^. Thus, it is likely that some methanotrophy occurs within these glacial rivers. However, the highly turbulent flow of the rivers promotes rapid methane outgassing^48^, which likely dominates over rate-limited methanotrophy.

### Subglacial geology and thermal conditions determine meltwater methane concentrations

**Fig. 4:**
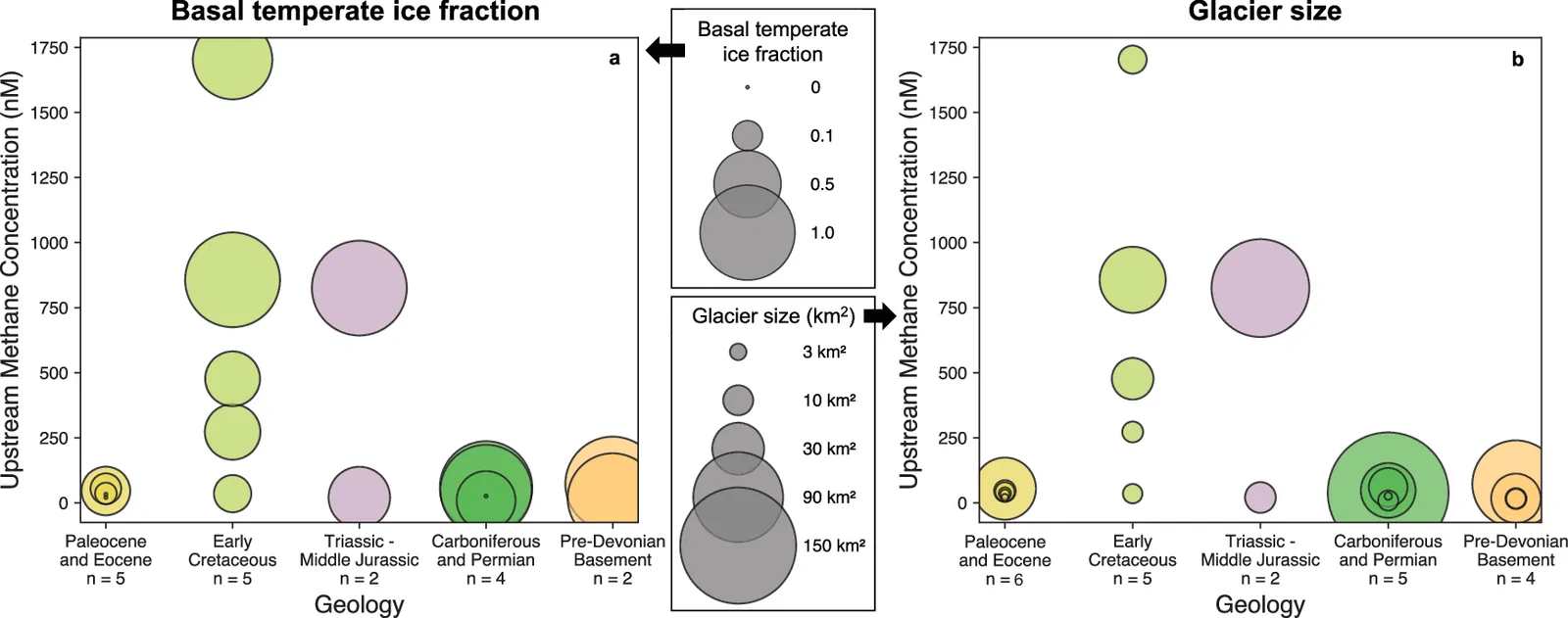
Basal temperate ice fractions and meltwater methane concentrations. Methane concentrations (nM) of the most upstream sample of each glacial river are plotted according to the main geological unit on which the glacier lies. Bubble size indicates the **a**, basal temperate ice fraction (*n* = 18), or the fraction of the bed that has temperate ice present or **b**, the area of the glacier in km^2^ (*n* = 22). The number of glaciers differs between the plots because GPR data was not available to calculate the basal temperate ice fraction for one glacier (Hessbreen (9)). Three glaciers (from the Ny-Ålesund region on Svalbard) were included in addition to the 19 glaciers in **b**, but these three glaciers were not included in the wider study because transect sampling was not conducted.

The glaciers selected for this study represent a range of sizes (0.56-110 km^2^), geological settings (Fig. 1), and thermal regimes. Our data reveal that the bedrock geology beneath the glaciers controls the potential for the melt river to contain high concentrations of methane. Statistical analysis shows that geology, when categorized into five units (as shown in Fig. 4), significantly predicts the log-transformed methane concentrations of upstream samples (analysis of variance, *F*_*4,13*_ = 3.50, *P* = 0.038, *n* = 18) and explains more than half of the variance in measured concentrations (*R*^2^ = 0.52, *n* = 18). Glaciers overlying shale-bearing geological formations, principally the Middle Triassic, Late Jurassic, and Early Cretaceous units on Svalbard, have the highest potential for methane-rich meltwaters emerging from their termini (Fig. 4).

However, the extent of methane supersaturation in the glacial outflows appears to be additionally governed by how hydrologically active the glacier bed is. We use the fraction of temperate ice (ice at or above its pressure melting point) at the base of the glacier (Fig. 4) as a proxy for how much of the bed is thawed and therefore hydrologically active. The presence of temperate ice at the glacier bed is strongly indicative of a thawed bed that is hydrologically connected to the drainage system. Glaciers with no temperate ice can be assumed with high certainty to be frozen to their bed, and thus there is little hydrological contact with geology^11–14^. When the basal temperature ice fraction is added to a statistical model and is considered in addition to the geology, they together explain three-quarters of the variance of log-transformed methane concentrations (analysis of covariance, *F*_*5,12*_ = 7.35, *P* = 0.0023, *R*^*2*^ = 0.75, *n* = 18). This indicates that the basal temperate ice fraction explains roughly one-quarter of the variance beyond geology.

**Fig. 5:**
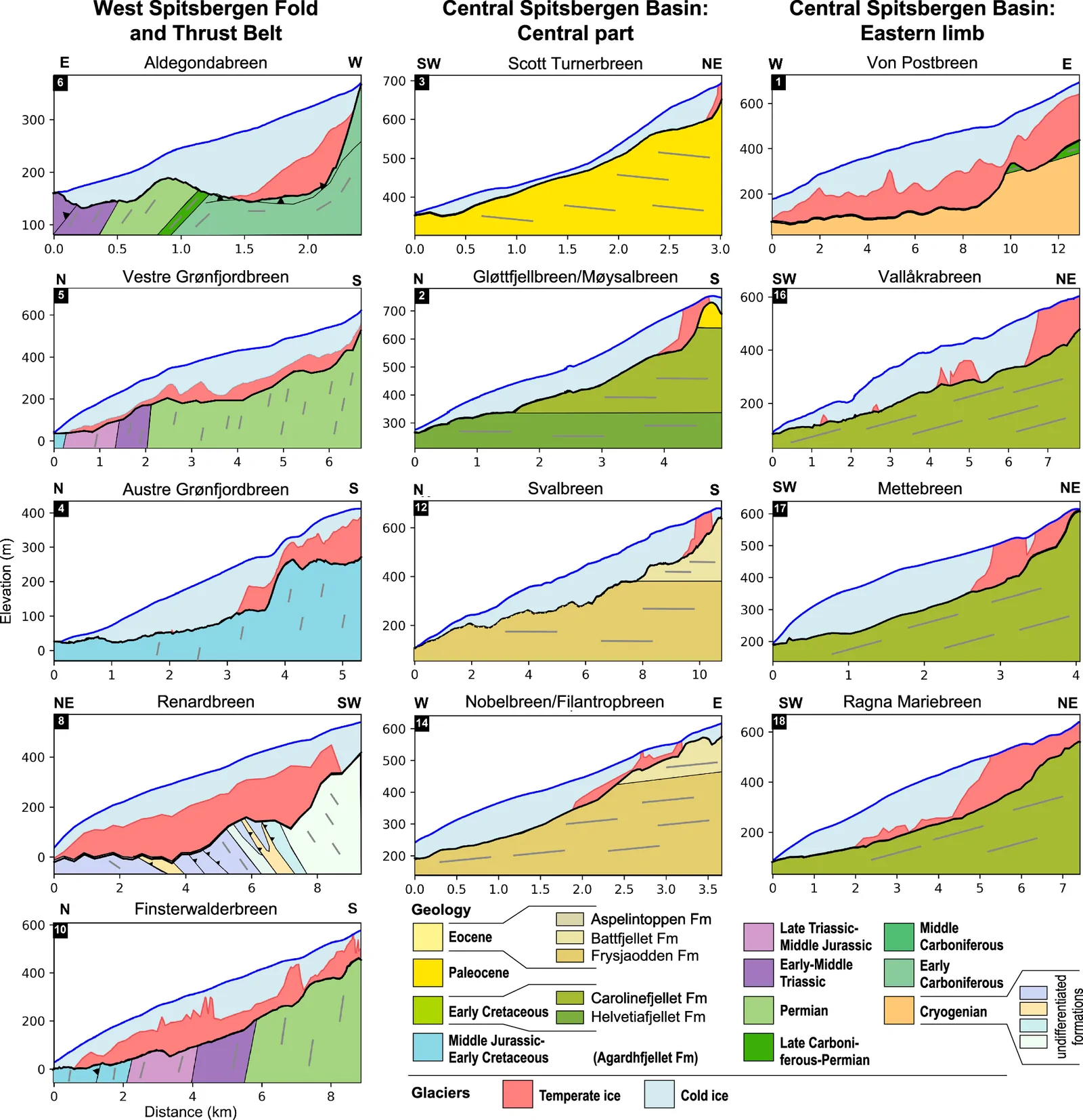
Glacier cross-sections and subglacial geology. Cross-sections of a selection of glaciers from this study with modeled subglacial geology and temperate ice zones measured by ground-penetrating radar (GPR)^66^. The lines in the geological units represent the dip angle. The Middle Triassic, Late Jurassic, and Early Cretaceous units contain abundant organic-rich shale layers. More detailed geological descriptions of each unit can be found in the Supplementary Information. Neighboring glaciers of similar sizes and geological settings were used as proxies in two instances when GPR surveys were not possible on the study glacier: Møysalbreen was used to represent Gløttfjellbreen (2), and Filantropbreen was used to represent Nobelbreen (14).

Our findings indicate that the bed must be thawed and hydrologically active where the glacier meets the shale-rich geology in order for the meltwater to effectively acquire methane. Thus, meltwaters flowing from a glacier above shale-rich rocks will likely contain high methane concentrations if temperate ice is present and the bed is thawed (e.g., Ragna Mariebreen (18) (1700 nM) in Fig. 5), but low concentrations if there is little or no temperate ice (ie., Gløttfjellbreen (2) (35 nM) in Fig. 5).

Thawed areas of the glacier bed can support methane supersaturation through several mechanisms. First, larger zones of unfrozen glacier bed enable greater water flow, supporting a more extensive subglacial drainage system that can flush methane stored in rocks, sediments and cavities below^18^. Second, methane-rich groundwaters may emerge at hydrologically active zones at the glacier bed and integrate into the subglacial drainage system^2^. Third, saturated, anoxic basal sediments provide ideal conditions for methanogenesis if any organic substrates are available^49^. On Svalbard, the first two mechanisms are evidently more significant, as we observe that glaciers with hydrologically active beds situated over geology lacking organic-rich strata—such as carbonate or crystalline bedrocks—exhibit low methane concentrations in their outflows, despite offering suitable conditions for methanogenesis. For example, Fig. 5 shows that Von Postbreen (1) and Renardbreen (8) are largely or entirely hydrologically active at their beds, yet their outflows contain relatively low methane concentrations (72 nM and 17 nM, respectively) because they lie on metamorphosed basement rocks.

Our findings suggest that as glacier melt continues to accelerate worldwide^50^, larger volumes of meltwater will be produced, increasing the amount of water reaching glacier beds and enhancing the flux of methane mobilized from subglacial environments. We also find evidence that glacier surge events, which are becoming more frequent on Svalbard^51^, may increase subglacial methane mobilization. Three of our rivers with the highest methane concentrations were actively surging or had recently finished a surge at the time of sampling (Penckbreen (11): 825 nM, Vallåkrabreen (16): 477 nM, Bakaninbreen (19): 857 nM). Sapper et al. suggested that subglacial methane production could be enhanced by glacier surging in Yukon, Canada, due to the remobilization of sediments supplying fresh organic material^17^. Here, we suggest that increased basal water pressures and expansion of the hydrologically active zone due to glacier surging^52,53^ enhances the flushing of subglacial methane stores.

On Svalbard, observations indicate that as valley glaciers recede and thin in a warming climate, they undergo a thermal transition from polythermal to cold-based systems^54^. During this transition, the proportion of thawed bed decreases until the glacier becomes entirely frozen to its bed^55,56^. As a result, methane fluxes from glacier meltwaters are likely to increase for some time, driven by enhanced subglacial hydrological activity, but may eventually decline as glaciers shift to cold-based systems where their capacity to mobilize and transport subglacial methane is limited.

Several glaciers in this study have likely reached this point, such as Sagabreen (7) and Bessemerbreen (14). Others, like Gløttfjellbreen (2), may be nearing this transition, which is reflected in low upstream methane concentrations in its melt river, despite its location within the shale-rich Early Cretaceous geological units. However, intra-permafrost groundwaters remain an effective pathway for delivering subsurface methane in these glacial catchments. This is evident across nearly all the rivers in this study, where abrupt increases in methane concentrations downstream from the glacier terminus suggest contributions from groundwater inputs (Fig. 4). Notably, peak methane concentrations occur far downstream from the glacier terminus in 68% of the rivers, indicating that the methane flux from groundwater is potentially more critical than the methane delivered by the subglacial drainage system. This is particularly relevant for glacial catchments in more advanced stages of deglaciation, like Gløttfjellbreen, where recently exposed forefields likely contain discontinuous permafrost^57^, allowing for intra-permafrost groundwater flow to sustain methane transport^2^. This finding highlights an important consideration for studies of methane release by glacial systems, which typically concentrate only on water emerging at the glacier terminus and may overlook important methane sources downstream.

### Potential emissions of methane from glacial rivers

We use our findings to provide a rough estimate of annual subglacial methane export from beneath each glacier by assessing the flux of methane emerging with the melt river at each glacier terminus. By combining the methane concentrations at the glacier terminus of each river with modeled total annual runoff for each glacier^58^, we estimate that the methane transport from beneath the glaciers ranges from 1 kg CH_4_ a^-1^ (0.9–1.1 kg CH_4_ a^-1^) for Bessemerbreen (13) to 5130 kg CH_4_ a^-1^ (4920-5350 kg CH_4_ a^-1^) for Penckbreen (11). The total methane transport for each glacier (mean: 416 kg a^-1^, median: 27 kg a^-1^) is detailed in Supplementary Table 1. Only one other study has quantified methane export from glacial melt rivers on Svalbard, where seasonal monitoring of the river showed that 618 kg CH_4_ (486–768 kg) were transported from beneath the Vallåkrabreen glacier during the 2021 melt season^18^. Our estimate for methane export from Vallåkrabreen (16), which is up to 607 kg CH_4_ a^-1^ (580–632 kg CH_4_ a^-1^), aligns closely with these previous findings, providing confidence in the robustness of our approach.

To approximate the scale of methane transport from beneath glaciers across Svalbard, we upscale our calculations using two methods. The first method applies a discharge-weighted-mean methane concentration to the total modeled land-terminating glacial runoff volume^58^ for Svalbard, yielding an estimate of 368 t CH_4_ a^-1^ (353-383 t CH_4_ a^-1^) in 2023 and 364 t CH_4_ a^-1^ (349-380 t CH_4_ a^-1^) in 2024. The second method uses a glacier-area-weighted-mean mass flux of methane, which is then applied to the total surface area of land-terminating glaciers on Svalbard^22^, resulting in estimates of 182 t CH_4_ a^-1^ (168-198 t CH_4_ a^-1^) in 2023 and 219 t CH_4_ a^-1^ (202-238 t CH_4_ a^-1^) in 2024. These projections are likely conservative, as they are based on methane concentrations measured at the most upstream sampling points, which were not always directly at the glacier terminus. Therefore, we do not account for methane lost from the river upstream of the termini in low-pressure channels or between the termini and the sampling locations. Additionally, our sampling was conducted primarily during mid-summer, which does not capture the early-season pulse of methane that has been stored beneath glaciers during winter and is flushed out at the onset of the melt season. This early-season pulse can represent a substantial portion of the total melt season methane flux^18^.

The methane transported by these rivers becomes available for emission to the atmosphere once it reaches the proglacial area. However, our isotopic measurements indicate that some methane may be oxidized in stream, meaning not all the methane is released directly to the atmosphere. Quantifying the extent of this oxidation is challenging with our current data because in-stream microbial methane production and inputs of methane-rich groundwater between sampling points further alter methane concentrations and isotopic compositions. While methane oxidation rates in rivers vary widely across studies^20,48,59^, it is generally observed that the rate of methane outgassing from rivers far exceeds the rate of microbial oxidation^48^, especially in shallow rivers with high surface-area-to-volume ratios, such as those in this study. This suggests that while some oxidation may occur, the majority of methane transported by these rivers is likely emitted to the atmosphere.

Our upscaled Svalbard-wide methane flux estimate is a similar order of magnitude to the lower-end estimate for methane emissions from glacial rivers across Greenland (918-2110 t CH_4_ yr^-1^)^60^. Given that Greenland's ice cover is more than 55-times larger than that of Svalbard, the flux per unit area is substantially greater in Svalbard. This disparity reflects the contrasting origins of methane between the two regions. Subglacial methane beneath Greenland’s glaciers is of microbial origin, produced in situ from organic material overridden during past glacial advances^60^. In contrast, Svalbard’s shale and carbon-rich geology supersaturates subglacial meltwaters with ancient thermogenic methane, which overwhelms any potential microbial source. This geologic source is evidently more effective at supersaturating meltwaters and thus yields disproportionately higher methane fluxes per unit area. These results implicate other glaciated regions that are situated within significant hydrocarbon-rich geological provinces, such as parts of Arctic Russia, Canada, northeast Greenland, and sections of Antarctica^5,61^, where glacial methane emissions have yet to be investigated.

Our calculated methane flux ranges are approximately an order of magnitude lower than the projected annual methane emissions from proglacial groundwater across Svalbard, which are estimated to be up to 2310 t CH_4_ per year^2^. Therefore, emissions from terrestrial seepage points of methane-rich groundwaters, such as those found downstream in glacial forefields^2^ and pingos^36^, appear to play a more critical role in contributing to the Arctic methane budget. This is an important consideration for future studies of methane release from glacial environments, as larger methane sources may exist downstream of the glacier terminus.

**Fig. 6:**
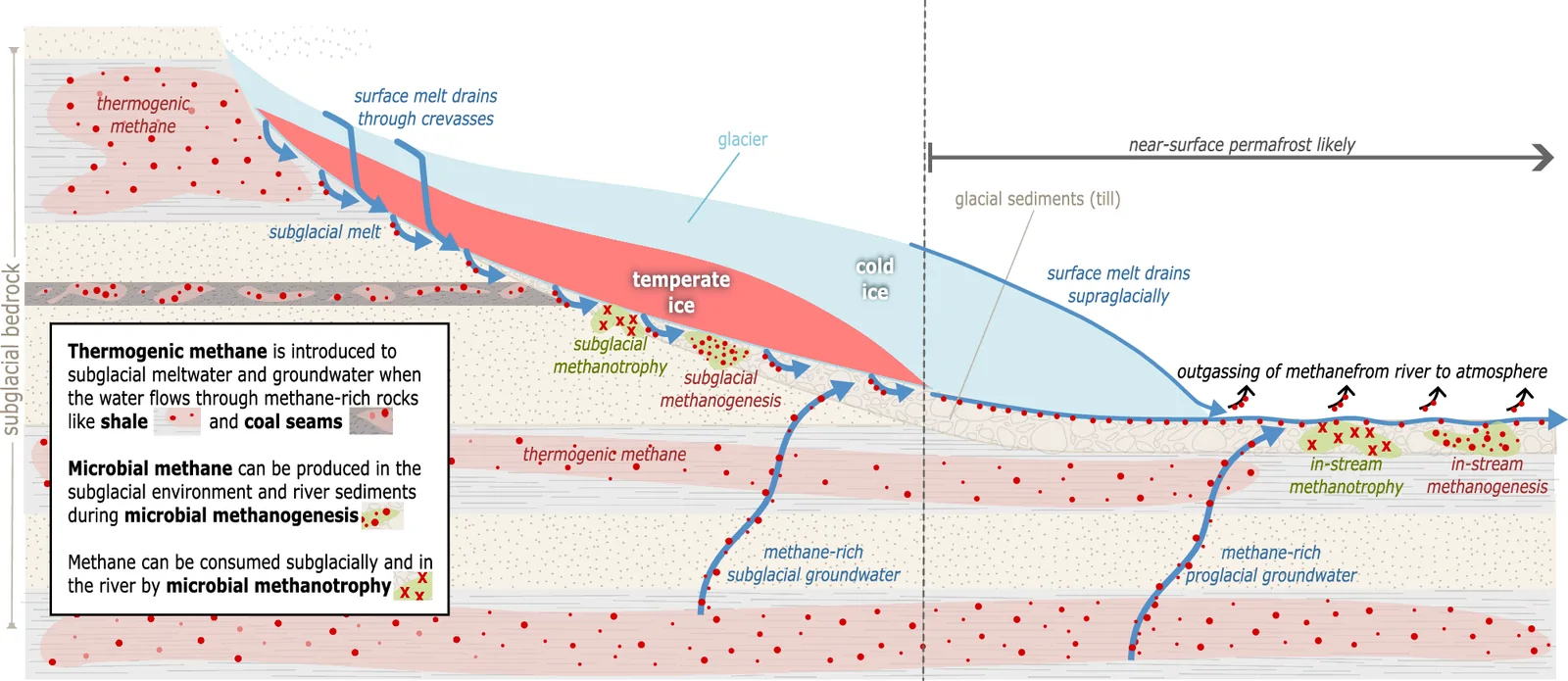
Conceptual model of subglacial methane mobilization. Methane can be introduced to the subglacial drainage system by thermogenic and microbial sources, especially beneath temperate ice zones where the ice-bed interface is thawed and hydraulically conductive. Permafrost beneath cold ice zones, where the ice-bed interface is frozen, substantially reduces hydraulic permeability, and therefore methane sources are limited. Once the glacial river is exposed to the atmosphere, methane begins to outgas, although some methane may be consumed in the river by in-stream methanotrophy. Potential additional sources of methane to the proglacial river include in-stream methanogenesis and inflows of methane-rich groundwater.

Our study demonstrates that the transport of subglacial methane by glacial rivers on Svalbard is governed by the underlying geology and the thermal regime of the glacier bed. Glaciers situated on shale-rich geological formations with thawed, hydrologically active beds mobilize significantly larger fluxes of methane. Subglacial methane on Svalbard is predominantly thermogenic in origin, as corroborated by isotopic signatures and wetness ratios of dissolved gases. While microbial processes such as methanogenesis and methanotrophy are evident in some systems, their contributions are secondary to the dominant geologic influence. Figure 6 summarizes the processes that mobilize, produce, and transform methane throughout the glacial environment.

As glaciers worldwide continue to melt in response to rising atmospheric temperatures^50^, the increased hydrological activity at glacier beds is likely to enhance methane mobilization. However, as glaciers thin and transition to cold-based systems, methane fluxes from subglacial drainage systems are expected to decline over time, leaving methane-rich groundwaters as the prevailing methane source. This study provides a framework for assessing potential methane fluxes from valley glaciers globally, particularly in regions where glaciers override organic-rich geology. These findings support a growing understanding of the importance of rivers, worldwide, in routing ancient carbon from land to the atmosphere^62^ and are critical for improving projections of natural methane emissions in a warming climate.

## Methods

### River transect sampling and analyses

River water samples were taken in transects along the length of 19 glacier melt rivers across central Svalbard, with two or three replicate samples taken at each site. The glacier catchments surveyed in this study were selected due to their accessibility in summer and are detailed in Fig. 1a and Supplementary Table 1. The most upstream samples were taken as close to the glacier front or subglacial portal as possible, which was typically less than 250 m downstream; however, in some cases was up to 1300 m downstream. The distance from the terminus of the first transect sample of each river is listed in Supplementary Table 1. The downstream samples, which ranged from 4 to 9 additional samples depending on the length of the river, were taken at relatively evenly spaced intervals until the river reached the mouth of its valley or flowed into the fjord. Unfiltered samples for methane concentration analysis were taken directly from the river at approximately 30 cm depth in 22 mL gas-tight serum vials. To prevent microbial activity during storage, samples were fixed with 1 mL of 1 M NaOH while simultaneously removing 1 mL of sample. This was done immediately after sampling. Samples for the analysis of the carbon isotopic composition of the methane, ethane, and propane concentrations were taken similarly, but in 60 mL gas-tight serum vials and fixed with 1 mL 37% HCl. All samples were stored upside down in the dark at ~4 °C until analysis. Samples were taken alongside in situ measurements of electrical conductivity, pH, and dissolved oxygen, which are reported in Supplemental Data 1.

To prepare samples for methane concentration analysis, a 4 mL headspace of pure N_2_ gas was added to each vial before being left to equilibrate at room temperature for at least 24 h. The concentration of methane in the headspace was measured at the University of Tromsø, Norway, by manual injection into a gas chromatograph (Thermo Scientific Trace 1310 GC) equipped with a Restek MSieve 5 A column and FID detector. The aqueous concentrations of methane in the original samples were calculated using the ideal gas law and solubility coefficients from ref. ^63^, as described in ref. ^2^. Methane concentration measurements were averaged among replicate samples and were within an analytical error of ±2.2%, calculated as the coefficient of variation of repeat measurements of 100 ppm standards (*n* = 6).

The stable carbon isotopic composition of methane (δ^13^C-CH_4_) in the river samples with methane concentrations greater than 25 nM were analyzed at the Federal Institute for Geosciences and Natural Resources, Hanover, Germany. Isotopic signatures were determined by a cryo-focusing technique (described in ref. ^64^) with a GC-C II/III (combustion interface) coupled to a Thermo Fisher Scientific MAT 253. All δ^13^C-CH_4_ measurements are reported relative to the Vienna Pee Dee Belemnite (VPDB) standard and with an analytical error of ±0.7‰ based on daily measurements of δ^13^C in air. Due to post-sampling analytical requirements, a combination of δ^13^C-CH_4_ measurements of acidified and alkalized samples was applied to determine the isotopic composition of dissolved methane along the transects of Von Postbreen (1), Scott Turnerbreen (3), Penckbreen (11), Svalbreen (12), and Finsterwalderbreen (10).

The ethane (C_2_H_6_) and propane (C_3_H_8_) concentrations of some river samples were measured to calculate corresponding wetness values (C_1_/(C_2_ + C_3_)). A 4.6 mL helium headspace was made in each sample vial and left to equilibrate at room temperature for at least two hours. Headspace concentrations were measured using a gas chromatography system (Trace 1310 GC, Thermo Fisher Scientific, USA) equipped with three heated sample loops and a valve system with column switching. Total pressure in the system was measured using a Keller pressure transducer (PAA-25, range 0-200 kPa, 0.4 kPa accuracy) before injection, and total headspace pressure was calculated from the sample loop pressures. The individual gas components (methane, ethane, and propane) were quantified in parallel on three separate channels. Aqueous concentrations were calculated using the partial pressures, sample temperatures, and Henry’s Law constants listed by ref. ^65^. Further details are provided in the Supplementary Information of ref. ^18^. The corresponding wetness values (C_1_/(C_2_ + C_3_)) were then calculated.

### Measurement of glacier area

The most up-to-date area of each glacier was measured on Sentinel-2 satellite images, which were taken within a week of each sampling date. The polygon feature in the geographic information system application QGIS was used to make three repeat area measurements, and an average measured area was used. Glacier areas are reported with an error of 4.0% based on the standard deviation of repeat measurements.

### Measurement of temperate ice and basal temperate ice fraction

Surface-coupled Ground Penetrating Radar (GPR) surveys were performed to identify the glacier bed and regions of temperate ice. New GPR surveys were carried out along the centerlines of Vallåkrabreen (16), Mettebreen (17), Ragna Mariebreen (18), Scott Turnerbreen (3), Finsterwalderbreen (10), Møysalbreen (as a proxy for Gløttfjellbreen (2)) and Filantropbreen (as a proxy for Nobelbreen (14)). These data are published in ref. ^66^. The proxy glaciers are similar in size to their representative glaciers and are in the same elevation ranges and geological settings, and are within a distance of 2 km from their representative glaciers. In addition, previously published raw GPR data from Svalbreen (12)^67^ was processed in the same way as the newly collected data. The surveys were carried out with a Malå ProEx GPR system using antennas with center frequencies of 25 MHz and 100 MHz, dragged by a snowmobile in spring. The data were post-processed using time-zero correction, dewow, bandpass Butterworth, and automatic gain correction in the software rsgpr version 0.3.3 (https://github.com/erikmannerfelt/rsgpr). A fixed medium velocity 0.168 m ns^-1^ was used for time to depth conversion^68^. The basal temperate ice fraction was calculated as the fraction of the glacier bed that has temperate ice present above it. Temperate ice is used as a proxy for thawing and hydrological connection of the glacier bed^15^.

Glacier thickness and the extent of temperate ice were provided from previously published GPR data on Renardbreen (8)^68^, Vestre Grønfjordbreen (5)^69^, Austre Grønfjordbreen (4)^69^, Aldegondabreen (6)^69^, and Von Postbreen (1)^70^. Glaciers without GPR data were dealt with in the following ways: Glaciers under 1 km² were assumed to have 0% basal temperate ice (Hettebreen (15), Sagabreen (7), Bessemerbreen (13)) and therefore frozen to their beds. Glaciers undergoing a surge or recently finished a surge (Penckbreen (11), Bakaninbreen (19)) were assumed to have a fully thawed glacier bed, equivalent to 100% basal temperate ice.

### Statistical analysis

Statistical analysis was performed in RStudio (version 2025.09.1 + 401). First, we tested the effect of bedrock geology on methane concentrations of our upstream water samples using a one-way analysis of variance (one-way ANOVA). Normality and variance equality were confirmed by visual inspection, and residual plots were used to confirm the fulfillment of relevant test assumptions. Methane concentrations were log_10_-transformed to meet these requirements. Next, we tested the combined effect of bedrock geology and basal temperate ice fraction on log_10_-transformed methane concentrations using an analysis of covariance (ANCOVA).

### Glacier and subglacial geology cross-sections (Fig. 4)

Subglacial geology was mapped using spatial correlation of published geological maps, cross-sections^71^ and digital terrain models^72^ with the glacier profiles. All relevant data were integrated in the Svalbox database using the Petrel 2022 Software^73^. Formation boundaries were digitized as surfaces using their intersections with the digital terrain models as input. These surfaces, and intersecting faults where present, were then projected on the glacier profiles to populate a geologically robust subglacial profile.

### Calculations of methane fluxes

The potential yearly flux of methane from each glacial river (*F*_*x*_, kg yr^-1^), as reported in Supplementary Table 1, was calculated using Eq. 1:

1 $${F}_{x}={[{{{{\rm{CH}}}}}_{4}]}_{T1,x}\times {Q}_{x}\times a\times {10}^{3}\times b$$

where [CH_4_]_T1,x_ is the methane concentration (nM) of the first (most upstream) sampling point of glacial river *x*, *Q*_*x*_ is the total discharge volume of glacial river *x* for the year the transect was sampled (m^3^ yr^-1^, derived from ref. ^58^), *a* is the conversion factor to convert nM to kg L^-1^ (1.604 ×10^-11^), 10^3^ converts m^3^ of river water to L, and *b* is the conversion factor to convert s^-1^ into yr^-1^ (3.1556736 ×10^7^). Error ranges are reported based on a ± 2.2% analytical error for measured methane concentrations and a ±2% error for modeled annual discharge volumes.

The annual methane flux from glacier melt rivers across all of Svalbard was crudely estimated with two methods to obtain a range of potential flux estimates. The first method was based on glacial runoff volumes and used the discharge-weighted-mean of the most upstream sampling points of each glacial river in this study. The mean concentration was multiplied by the total terrestrial glacier runoff across the entire Svalbard archipelago, which is modeled by ref. ^58^. Values were calculated with both the 2023 and 2024 total runoff estimates. The second method was based on glacier area and used the glacier-area-weighted mean of the methane fluxes from all 19 glaciers. This mean flux was multiplied by the total area of land-terminating glaciers on Svalbard (10 808 km^2^), as reported in ref. ^22^. Error ranges are reported based on a ± 2.2% analytical error for measured methane concentrations, ±2% error for modeled annual discharge volumes and ±4.0% error for measured glacier areas.

## Supplementary information

Supplementary Information

Peer Review file

Description of Additional Supplementary Files

Supplementary Data 1

## Data Availability

The hydro-chemical data generated in this study are provided in the Supplementary Information and Supplementary Data 1 files.

## References

[1] Rantanen, M. et al. The Arctic has warmed nearly four times faster than the globe since 1979. *Commun. Earth Environ.* **3**, 1–10 (2022).

[2] Kleber, G. E. et al. Groundwater springs formed during glacial retreat are a large source of methane in the high Arctic. *Nat. Geosci.* **16**, 597–604 (2023).

[3] Schuur, E.aG. et al. Climate change and the permafrost carbon feedback. *Nature* **520**, 171–179 (2015). 25855454 10.1038/nature14338

[4] Meredith, M. et al. *The Ocean and Cryosphere in a Changing Climate: Special Report of the Intergovernmental Panel on Climate Change*. 203–320 https://www.cambridge.org/core/product/identifier/9781009157964/type/book(2019).

[5] Gautier, D. L. et al. Assessment of Undiscovered Oil and Gas in the Arctic. *Science* **324**, 1175–1179 (2009). 19478178 10.1126/science.1169467

[6] Patton, H. et al. The extreme yet transient nature of glacial erosion. *Nat. Commun.* **13**, 7377 (2022). 36450722 10.1038/s41467-022-35072-0 PMC9712427

[7] Vachon, R. et al. Glacially induced stress across the arctic from the eemian interglacial to the present—implications for faulting and methane seepage. *J. Geophys. Res. Solid Earth* **127**, e2022JB024272 (2022).

[8] Wadham, J. L., Tranter, M., Tulaczyk, S. & Sharp, M. Subglacial methanogenesis: a potential climatic amplifier?. *Glob. Biogeochem. Cycles* **22**, GB2021 (2008).

[9] Walter Anthony, K. M., Anthony, P., Grosse, G. & Chanton, J. Geologic methane seeps along boundaries of Arctic permafrost thaw and melting glaciers. *Nat. Geosci.* **5**, 419–426 (2012).

[10] Portnov, A., Vadakkepuliyambatta, S., Mienert, J. & Hubbard, A. Ice-sheet-driven methane storage and release in the Arctic. *Nat. Commun.* **7**, 10314 (2016). 26739497 10.1038/ncomms10314 PMC4729839

[11] Christiansen, H. H., French, H. M. & Humlum, O. Permafrost in the Gruve-7 mine, Adventdalen, Svalbard. *Nor. Geografisk Tidsskr. - Nor. J. Geogr.* **59**, 109–115 (2005).

[12] King, E. C. Glacier bed characteristics of Midtre Lovénbreen, Svalbard, from high-resolution seismic and radar surveying. *J. Glaciol.* **54**, 145–156 (2008).

[13] Kulessa, B. & Murray, T. Slug-test derived differences in bed hydraulic properties between a surge-type and a non surge-type Svalbard glacier. *Ann. Glaciol.* **36**, 103–109 (2003).

[14] Irvine-Fynn, T. D. L., Hodson, A. J., Moorman, B. J., Vatne, G. & Hubbard, A. L. Polythermal glacier hydrology: a review. *Rev. Geophys.* **49**, RG4002 (2011).

[15] Gilbert, A. et al. The influence of water percolation through crevasses on the thermal regime of a Himalayan mountain glacier. * Cryosphere* **14**, 1273–1288 (2020).

[16] Lamarche-Gagnon, G. et al. Greenland melt drives continuous export of methane from the ice-sheet bed. *Nature* **565**, 73–77 (2019). 30602750 10.1038/s41586-018-0800-0

[17] Sapper, S. E., Jørgensen, C. J., Schroll, M., Keppler, F. & Christiansen, J. R. Methane emissions from subglacial meltwater of three alpine glaciers in Yukon, Canada. *Arct. Antarct. Alp. Res.* **55**, 2284456 (2023).

[18] Kleber, G. E. et al. Proglacial methane emissions driven by meltwater and groundwater flushing in a high-Arctic glacial catchment. *Biogeosciences* **22**, 659–674 (2025).

[19] Burns, R. et al. Direct isotopic evidence of biogenic methane production and efflux from beneath a temperate glacier. *Sci. Rep.* **8**, 17118 (2018). 30459433 10.1038/s41598-018-35253-2 PMC6244297

[20] Strock, K. E., Krewson, R. B., Hayes, N. M. & Deemer, B. R. Oxidation is a potentially significant methane sink in land-terminating glacial runoff. *Sci. Rep.* **14**, 23389 (2024). 39379398 10.1038/s41598-024-73041-3 PMC11461896

[21] Christiansen, J. R., Röckmann, T., Popa, M. E., Sapart, C. J. & Jørgensen, C. J. Carbon emissions from the edge of the greenland ice sheet reveal subglacial processes of methane and carbon dioxide turnover. *JGR Biogeosci.* **126**, e2021JG006308 (2021).

[22] Nuth, C. et al. Decadal changes from a multi-temporal glacier inventory of Svalbard. * Cryosphere* **7**, 1603–1621 (2013).

[23] Senger, K. et al. Geology of Svalbard: Deep-time and Deep-Earth. *SESS Rep.* **2024**, 52–83 (2025).

[24] Hodson, A. *et al*. *Methane in Svalbard (SvalGaSess)*. https://zenodo.org/doi/10.5281/zenodo.14425572(2025).

[25] Pirk, N. et al. Toward a statistical description of methane emissions from Arctic wetlands. *Ambio*. **46**, 70–80 (2017). 28116692 10.1007/s13280-016-0893-3 PMC5258667

[26] Noël, B. et al. Low elevation of Svalbard glaciers drives high mass loss variability. *Nat. Commun.* **11**, 4597 (2020). 32929066 10.1038/s41467-020-18356-1 PMC7490702

[27] Whiticar, M. J. Carbon and hydrogen isotope systematics of bacterial formation and oxidation of methane. *Chem. Geol.* **161**, 291–314 (1999).

[28] Adnew, G. A. et al. Clumped isotope measurements reveal aerobic oxidation of methane below the Greenland ice sheet. *Geochimica et. Cosmochimica Acta*. **389**, 249–264 (2025).

[29] Dieser, M. et al. Molecular and biogeochemical evidence for methane cycling beneath the western margin of the Greenland Ice Sheet. *ISME J.* **8**, 2305–2316 (2014). 24739624 10.1038/ismej.2014.59 PMC4992074

[30] Ohm, S. E. et al. Discovery of shale gas in organic rich Jurassic successions, Adventdalen, Central Spitsbergen, Norway. *Nor. J. Geol.* **99**, 349–376 (2019).

[31] Olaussen, S. et al. Svalbard composite tectono-sedimentary element, Barents Sea. *Geol. Soc., Lond., Mem.* **57**, M57-2021–36 (2025).

[32] Knies, J., Damm, E., Gutt, J., Mann, U. & Pinturier, L. Near-surface hydrocarbon anomalies in shelf sediments off Spitsbergen: evidences for past seepages. *Geochem. Geophys. Geosyst.* **5**, Q06003 (2004).

[33] Abay, T. B. et al. Migrated petroleum in outcropping Mesozoic sedimentary rocks in Spitsbergen: organic geochemical characterization and implications for regional exploration. *J. Pet. Geol.* **40**, 5–36 (2017).

[34] Rodes, N. *et al*. From Source Rocks to the Surface: The Fate of Gas Seepage in High Arctic Fjords, Svalbard. in vol. 2022 1–5 (European Association of Geoscientists & Engineers, 2022).

[35] Roy, S., Hovland, M., Noormets, R. & Olaussen, S. Seepage in Isfjorden and its tributary fjords, West Spitsbergen. *Mar. Geol.* **363**, 146–159 (2015).

[36] Hodson, A. J. et al. Sub-permafrost methane seepage from open-system pingos in Svalbard. * Cryosphere* **14**, 3829–3842 (2020).

[37] Milkov, A. V. & Etiope, G. Revised genetic diagrams for natural gases based on a global dataset of >20,000 samples. *Org. Geochem.* **125**, 109–120 (2018).

[38] Vinšová, P. et al. The Biogeochemical Legacy of Arctic Subglacial Sediments Exposed by Glacier Retreat. *Glob. Biogeochem. Cycles* **36**, e2021GB007126 (2022).

[39] Macdonald, M. L., Wadham, J. L., Telling, J. & Skidmore, M. L. Glacial erosion liberates lithologic energy sources for microbes and acidity for chemical weathering beneath glaciers and ice sheets. *Front. Earth Sci.* **6**, 212 (2018).

[40] Hall, A. & van Boeckel, M. Hydraulic damage in subglacial conduits: evidence from rock hydrofracture and hydraulic jacking for high fluid pressures during rapid melt of the Fennoscandian Ice Sheet. *Quat. Sci. Rev.* **343**, 108917 (2024).

[41] Phillips, E., Everest, J. & Reeves, H. Micromorphological evidence for subglacial multiphase sedimentation and deformation during overpressurized fluid flow associated with hydrofracturing. *Boreas* **42**, 395–427 (2013).

[42] Hodson, A. et al. Effects of glacier retreat upon glacier-groundwater coupling and biogeochemistry in Central Svalbard. *J. Hydrol.* **624**, 129894 (2023).

[43] Lupon, A. et al. Groundwater inflows control patterns and sources of greenhouse gas emissions from streams. *Limnol. Oceanogr.* **64**, 1545–1557 (2019).

[44] Chesnokova, A., Baraër, M. & Bouchard, É Proglacial icings as records of winter hydrological processes. * Cryosphere* **14**, 4145–4164 (2020).

[45] Knox, M., Quay, P. D. & Wilbur, D. Kinetic isotopic fractionation during air-water gas transfer of O2, N2, CH4, and H2. *J. Geophys. Res. Oceans* **97**, 20335–20343 (1992).

[46] Kleber, G. E. et al. Shallow and deep groundwater moderate methane dynamics in a high Arctic glacial catchment. *Front. Earth Sci.* **12**, 1340399 (2024).

[47] Znamínko, M. et al. Methylotrophic communities associated with a Greenland ice sheet methane release hotspot. *Microb. Ecol*10.1007/s00248-023-02302-x(2023). PMC10640400 37843656

[48] Rovelli, L. et al. Contrasting Biophysical Controls on Carbon Dioxide and Methane Outgassing From Streams. *J. Geophys. Res. Biogeosci.* **127**, e2021JG006328 (2022).

[49] Stibal, M. et al. Methanogenic potential of Arctic and Antarctic subglacial environments with contrasting organic carbon sources. *Glob. Change Biol.* **18**, 3332–3345 (2012).

[50] Hugonnet, R. et al. Accelerated global glacier mass loss in the early twenty-first century. *Nature* **592**, 726–731 (2021). 33911269 10.1038/s41586-021-03436-z

[51] Strozzi, T. et al. Glacier surge activity over Svalbard from 1992 to 2025 interpreted using heritage satellite radar missions and Sentinel-1. * Cryosphere* **20**, 1679–1697 (2026).

[52] Kamb, B. et al. Glacier surge mechanism: 1982-1983 surge of variegated glacier, Alaska. *Science* **227**, 469–479 (1985). 17733459 10.1126/science.227.4686.469

[53] Sevestre, H., Benn, D. I., Hulton, N. R. J. & Bælum, K. Thermal structure of Svalbard glaciers and implications for thermal switch models of glacier surging. *J. Geophys. Res. Earth Surf.* **120**, 2220–2236 (2015).

[54] Mannerfelt, E. S., Hodson, A., Håkansson, L. & Lovell, H. Dynamic LIA advances hastened the demise of small valley glaciers in central Svalbard. *Arct. Sci.* **10**, 815–833 (2024).

[55] Hambrey, M. J. et al. Structure and changing dynamics of a polythermal valley glacier on a centennial timescale: Midre Lovénbreen, Svalbard. *J. Geophys. Res. Earth Surf.* **110**, F01006 (2005).

[56] Lovell, H. et al. Former dynamic behaviour of a cold-based valley glacier on Svalbard revealed by basal ice and structural glaciology investigations. *J. Glaciol.* **61**, 309–328 (2015).

[57] Moorman, B. J. Glacier-permafrost hydrology interactions, Bylot Island, Canada. In *Proc. 8th International Conference on Permafrost* 783–788 (Wiley, 2003).

[58] Schmidt, L. S. & Schuler, T. V. Runoff simulations for Svalbard, 1991-2023. npolar.no 10.21334/NPOLAR.2024.87FA4A0A (2024).

[59] Heilweil, V. M., Solomon, D. K., Darrah, T. H., Gilmore, T. E. & Genereux, D. P. Gas-tracer experiment for evaluating the fate of methane in a coastal plain stream: degassing versus in-stream oxidation. *Environ. Sci. Technol.* **50**, 10504–10511 (2016). 27632066 10.1021/acs.est.6b02224

[60] Hatton, J. E. et al. Mid-Holocene retreat of the Greenland ice sheet indicated by subglacial methane release. *Nat. Geosci*. 1–7 10.1038/s41561-026-01976-5(2026).

[61] Wadham, J. L. et al. An ice sheet-to-ocean analysis of carbon stores and fluxes in Earth’s Polar Regions (RECCAP2, Polar Ice Sheets). *Glob. Biogeochem. Cycles* **40**, e2025GB008677 (2026).

[62] Dean, J. F. et al. Old carbon routed from land to the atmosphere by global river systems. *Nature* **642**, 105–111 (2025). 40467741 10.1038/s41586-025-09023-w PMC12137138

[63] Wiesenburg, D. A. & Guinasso, N. L. Equilibrium solubilities of methane, carbon monoxide, and hydrogen in water and sea water. *J. Chem. Eng. Data* **24**, 356–360 (1979).

[64] Schloemer, S., Elbracht, J., Blumenberg, M. & Illing, C. J. Distribution and origin of dissolved methane, ethane and propane in shallow groundwater of Lower Saxony, Germany. *Appl. Geochem.* **67**, 118–132 (2016).

[65] Sander, R. Compilation of Henry’s law constants (version 4.0) for water as solvent. *Atmos. Chem. Phys.* **15**, 4399–4981 (2015).

[66] Mannerfelt, E. S. et al. Glacier thickness, thermal regime, and subjective uncertainty from ground-penetrating radar of 25 Svalbard glaciers. *J. Glaciol.* (In Review) 10.5281/zenodo.21890778.

[67] Johansen, T. A. et al. Seismic profiling on Arctic glaciers. *First Break* **29**, 65–71 (2011).

[68] Navarro, F. J. et al. Ice volume estimates from ground-penetrating radar surveys, Wedel Jarlsberg Land Glaciers, Svalbard. *Arct. Antarct. Alp. Res.* **46**, 394–406 (2014).

[69] Macheret, Y., Glazovsky, A. & Lavrentiev, I. Distribution of cold and temperate ice and water in glaciers at Nordenskiöld Land, Svalbard, according to data on ground-based radio-echo sounding. *Bulletin of Geography. Physical Geography Series* 77–90 10.2478/bgeo-2019-0016(2019).

[70] Delf, R. et al. Reanalysis of Polythermal Glacier Thermal Structure Using Radar Diffraction Focusing. *J. Geophys. Res. Earth Surf.* **127**, e2021JF006382 (2022).

[71] Dallmann, W. K. Geoscience Atlas of Svalbard. *Norsk Polarinstitutt* https://digitalcommons.usf.edu/kip_articles/2176 (2015).

[72] Terrengmodell S. NPDC. https://data.npolar.no/dataset/dce53a47-c726-4845-85c3-a65b46fe2fea

[73] Senger, K. et al. Using digital outcrops to make the high Arctic more accessible through the Svalbox database. *J. Geosci. Educ.* **69**, 123–137 (2021).

