# Supplementary material for: Subglacial geology and thermal conditions regulate methane emissions from Svalbard glaciers
Source: Nat Commun. 2026 Sep 8;17:9347. doi: 10.1038/s41467-026-77190-z (PMC13554054; doi:10.1038/s41467-026-77190-z)
Supplement: Supplementary file 1 [file 41467_2026_77190_MOESM1_ESM.pdf]

Supplementary Information

Details of Svalbard Geology

Below is a list of the Svalbard geological units relevant to this study (from Fig. 4 in the main text), with more detailed explanations as described by Dallman (2015)[1].

Eocene: clastic sedimentary infill of the Central Tertiary Basin (CTB), erosional products of the West Spitsbergen Fold Belt; sandstones interbedded with minor shales, siltstones and thin coals; tidally-influenced delta-related shelf environments and coastal plains

Aspelintoppen Formation: alterations of sandstones, siltstones and shale

Battfjellet Formation: clinoform-shaped shelf margin made up of interfingering greenish sandstone wedges

Frysjaodden Formation: dark shales of shelf origin

Paleocene: clastic sedimentary infill of the CTB, sandstone unit underlain by coal-bearing strata interbedded with marine and non-marine sandstones, siltstones, shales and conglomerates; deltaic in origin

Early Cretaceous: shales, sandstones, siltstones and subordinate coals, conglomerates and rare bentonites

Carolinefjellet Formation: alternating sandstone- and shale-dominated units deposited in open-marine deep shelf environment

Helvetiafjellet Formation: lower sandstone- and conglomerate-dominated part and upper heterolithic part with sandstones, carbonaceous shales and thin coals; deposited in braided rivers and marginal marine and coastal-plain environments

Middle Jurassic-Early Cretaceous (Agardhfjellet Formation): shale-dominated with variable content of organic matter in siltstone/sandstone and minor carbonate beds and concretions, prolific hydrocarbon source rocks; deposited in inner shelf to offshore environments

Late Triassic-Middle Jurassic: uplifted parts of the Barents Sea shelf; shale and siltstone including hydrocarbon source rocks and sandstone reservoir rocks; shallow-marine to deltaic deposits

Early-Middle Triassic: marine clastics deposited in a low-relief shelf embayment with coastal to deltaic sediments; marine organic-rich shale dominated with siltstones, and sandstones; all hydrocarbon has already been expelled in Western Spitsbergen[2]

Permian: cherts, fossiliferous limestones and minor sandstones and black shales; deposited in open-marine shelf under temperate to cold-water conditions

Late Carboniferous-Permian: mixed clastic, carbonate and evaporite successions deposited in marginal to open marine conditions

Middle Carboniferous: conglomerates, sandstones, siltstones and shales deposited in fluvial to alluvial environments in a warm, arid to semi-arid climate

Early Carboniferous: clastic terrestrial deposits of sandstones and siltstones with minor conglomerates and shales with coal seams

Cryogenian: metasedimentary units with shale and carbonate beds and glacial diamictites deposited during a cold climate and originally moraines, glacifluvial deposits and dropstone deposits

Methods

Incubation of river and groundwater water

Two incubation experiments were done with water collected from the river at Svalbreen and an anoxic groundwater seep in the Svalbreen river plain (77.7324°N, 16.4982°E). Replicate individual samples were taken at the same time and location (16 at the river site and 16 at the groundwater site) by the same water sampling methods described in the main text. Samples were fixed with 1 mL of 1 M NaOH (1M) at timed intervals (0, 8, 30, 40h). At every timestep, three samples were fixed thus creating a triplicate incubation. A notable difference from standard incubations is that samples were separated from the start of the incubation. The samples were stored together in a 4 L plastic container filled with the source river water with a temperature logger measuring every 15 minutes. The temperature of the source water was 0.8°C. Temperature fluctuation could not be avoided in the field, resulting in an increase from 6 to 12°C over 22 hours and then a decrease to 2°C until 36 hours, after which samples were stored at a stable 6 °C in a fridge. Methane concentrations of the samples were analyzed by the same methods described in the main text.

Results: Figures and Tables

Incubations

The river incubation showed net methanogenesis, while the groundwater incubation showed net oxidation up until 30 hours. Both incubations showed a steep increase in methane concentrations from 30 to 40 hours. Both incubations had suspended sediments which inadvertently settled over time. Measurements after 30 hours are excluded, as the increased methane production is most likely a product of oxygen depletion and thus unrepresentative rates of methanogenesis in the settled sediments. The sharp increase in methane from 30 to 40 hours indicates that a methanogenic community was already established in both water sources, and methane production was ongoing in the suspended sediments within the otherwise aerobic water.

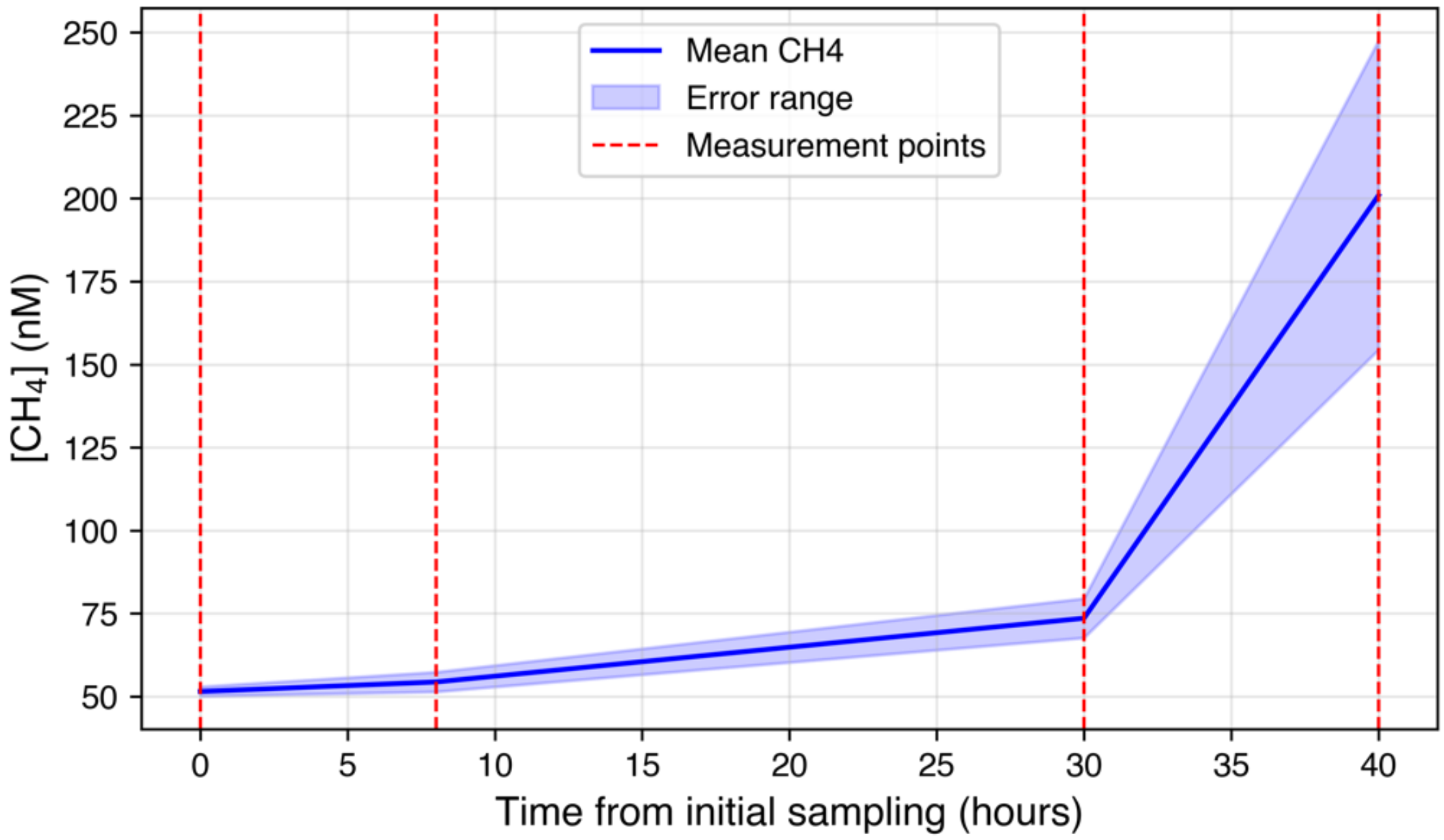

Supplementary Fig. 1. Incubation results from Svalbreen river water. The shaded error range represents the standard error of the mean.

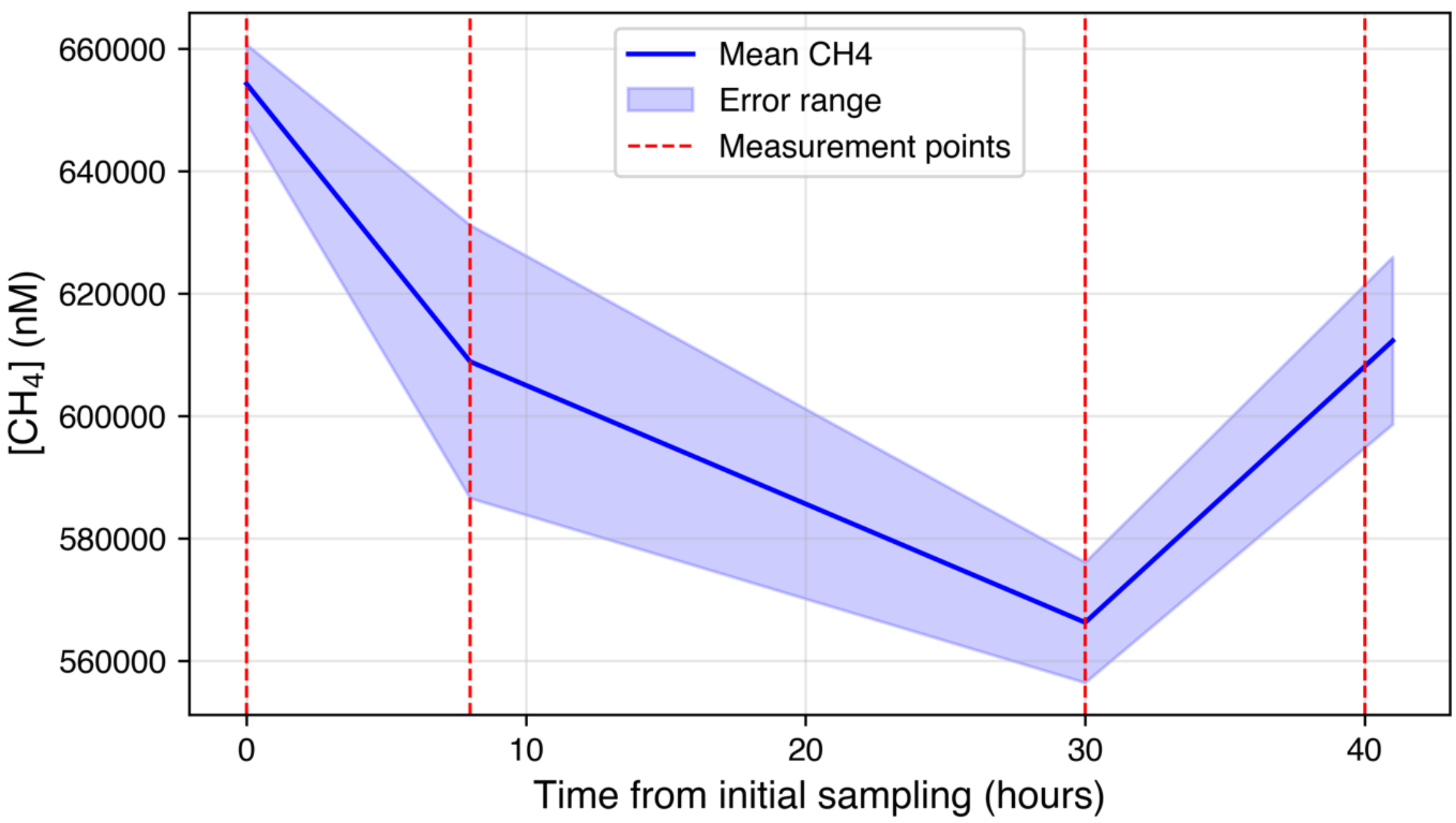

Supplementary Fig. 2. Incubation results from Svalbreen catchment groundwater. The shaded error range represents the standard error of the mean.

Supplementary Table 1. Summary of metadata and data acquired for each glacier.

Label from Fig. 1	Glacier Name	Coordinates of first transect sample	Date of sampling	First sample [CH_4] (nM)	First sample $\delta^{13}C$–CH_4 (‰)	First sample's distance from portal or glacier terminus (m)	Primary Geology[a]	Glacier Area (km^2)	Basal Temperate Ice Fraction	Total run-off 2023[b] (10^6 m^3 a^{-1})	Total run-off 2024[b] (10^6 m^3 a^{-1})	Total yearly CH_4 flux from river (2023)[c] (kg CH_4 a^{-1})	Total yearly CH_4 flux from river (2024)[c] (kg CH_4 a^{-1})
**1**	*Von Postbreen*	*78.41828°N, 17.52026°E*	*25-Jul-24*	*72*	*-43.7*	*44*	*5*	*85*	*1.000*	*203*	*218*	*235*	*252*
**2**	*Gløttfjellbreen*	*78.146673°N, 16.479620°E*	*23-Aug-24*	*35*	*-48.4*	*1215*	*2*	*4.2*	*0.155**	*14*	*16*	*8*	*9*
**3**	*Scott Turnerbreen*	*78.11180°N, 15.98857°E*	*02-Aug-24*	*36*	*-49.1*	*1352*	*1*	*1.7*	*0.052*	*11*	*12*	*6*	*7*
**4**	*Austre Grønfjordbreen*	*77.922429°N, 14.313234°E*	*09-Jul-24*	*20*	*-52.1*	*341*	*3*	*11*	*0.419*	*29*	*41*	*9*	*13*
**5**	*Vestre Grønfjordbreen*	*77.936983°N, 14.244057°E*	*10-Jul-24*	*60*	*-49.4*	*24*	*4*	*17*	*0.948*	*47*	*68*	*45*	*65*
**6**	*Aldegondabreen*	*77.97804°N, 14.09637°E*	*10-Jul-24*	*9*		*125*	*4*	*4.6*	*0.385*	*19*	*25*	*3*	*4*
**7**	*Sagabreen*	*77.777018°N, 14.426215°E*	*17-Aug-24*	*26*	*-38.7*	*212*	*4*	*0.6*	*0†*	*5*	*5*	*2*	*2*
**8**	*Renardbreen*	*77.535646°N, 14.470256°E*	*12-Jul-24*	*17*	*-47.8*	*19*	*5*	*27*	*0.906*	*87*	*112*	*24*	*30*
**9**	*Hessbreen*	*77.521611°N, 15.163609°E*	*19-Sep-23*	*42*	*-38.0*	*233*	*1*	*4.2*		*17*	*22*	*12*	*15*
**10**	*Finsterwalderbreen*	*77.506426°N, 15.287862°E*	*14-Jul-24*	*48*	*-35.6*	*180*	*4*	*33*	*0.928*	*116*	*158*	*89*	*121*
**11**	*Penckbreen*	*77.512416°N, 15.650103°E*	*13-Jul-24*	*825*	*-40.4*	*0*	*3*	*106*	*1‡*	*305*	*389*	*4026*	*5130*
**12**	*Svalbreen*	*77.708007°N, 16.557585°E*	*18-Aug-24*	*54*	*-54.1*	*670*	*1*	*43*	*0.105*	*83*	*96*	*73*	*83*
**13**	*Bessemerbreen*	*77.790857°N, 16.672534°E*	*27-Jul-23*	*21*		*265*	*1*	*0.6*	*0†*	*3*	*3*	*1*	*1*
**14**	*Nobelbreen*	*77.758256°N, 16.753440°E*	*26-Jul-24*	*46*	*-52.4*	*0*	*1*	*5.2*	*0.263**	*18*	*20*	*13*	*14*
**15**	*Hettebreen*	*77.755288°N, 16.782886°E*	*17-Aug-23*	*33*	*-49.6*	*439*	*1*	*1.0*	*0†*	*4*	*4*	*2*	*2*
**16**	*Vallåkrabreen*	*77.832690°N, 17.059602°E*	*19-Jul-23*	*477*	*-46.0*	*12*	*2*	*19*	*0.334*	*70*	*80*	*537*	*607*
**17**	*Mettebreen*	*77.812248°N, 17.211187°E*	*18-Jul-23*	*272*	*-41.6*	*184*	*2*	*4.8*	*0.343*	*11*	*13*	*47*	*57*
**18**	*Ragna Mariebreen*	*77.774029°N, 17.281271°E*	*14-Jul-23*	*1703*	*-42.8*	*37*	*2*	*8.8*	*0.702*	*21*	*22*	*582*	*590*
**19**	*Bakaninbreen*	*77.772965°N, 17.235756°E*	*04-Aug-24*	*857*	*-46.0*	*37*	*2*	*49*	*1‡*	*107*	*118*	*1464*	*1618*
	Kongsbreen ˣ	*78.85631°N, 12.55949°E*	*15-Jul-24*	*38*			*4*	*163*					
	Pedersenbreen ˣ	*78.8792°N, 12.3061°E*	*16-Jul-24*	*16*			*5*	*4.9*					
	Feiringbreen ˣ	*79.0057°N, 12.4568°E*	*17-Jul-24*	*16*			*5*	*4.3*					

[a] *1. Eocene, 2. Middle Jurassic, 3. Triassic, 4. Permian, 5. Neoproterozoic*

[b] *Runoff from ref. 59*

[c] *Speculative estimation calculated from first transect sample methane concentration and total modelled runoff*

ˣ *No transect sampling was conducted, only upstream. Data only included in Fig. 3b of the main text.*

* *The basal temperate ice fraction of Møysalbreen and Filantropbreen have been used as a proxy for Gløttfjellbreen and Nobelbreen, respectively.*

† *Assumed to be 0 due to the glacier area being < 1 km^2*

‡ *Assumed to be 1 due to the glacier presently surging or having recently surged*
