# Supplementary material for: Subglacial geology and thermal conditions regulate methane emissions from Svalbard glaciers
Source: Nat Commun. 2026 Sep 8;17:9347. doi: 10.1038/s41467-026-77190-z (PMC13554054; doi:10.1038/s41467-026-77190-z)
Supplement: Supplementary file 2 [file 41467_2026_77190_MOESM2_ESM.pdf]

nature portfolio

Peer Review File

Ice, shale and methane: linking glacier bed conditions to Arctic methane release

Corresponding Author: Dr Gabrielle Kleber

This file contains all reviewer reports in order by version, followed by all author rebuttals in order by version.

Version 0:

Reviewer comments:

Reviewer #1

(Remarks to the Author)
The authors collected CH4 concentration data near the outflows of 19 Svalbard glaciers, plus additional downstream samples along the length of rivers as they flowed away from the glaciers. The region is known for its hydrocarbon-rich lithology, which serves as a source of CH4 in the study rivers. The main idea of this paper is that the temperate ice fraction controls the concentration of CH4 in glacier meltwater streams. The implication is that future warming will increase the temperate ice fraction, mobilizing and releasing to the atmosphere more geologic CH4.
For clarity, my comments will use the meltwater channel as the point of reference when terms ‘recharge’ and ‘discharge’ are used. Recharge means that the river is gaining water, while discharge means that it is losing water to the subsurface groundwater reservoir.
My main concerns with the manuscript are insufficient understanding and explanation of sub-glacial hydrology and lack of support for the proposed mechanism of temperate ice fraction control on CH4 concentration.
The interpretation of the data collected is based on a conceptual model (Fig. 5), but details of this model are not described in the text or supported by the observations. For instance, (a) terms such ‘high-pressure drainage’ and ‘low-pressure drainage” appear to be part of the proposed mechanism of meltwater entrainment and transport of geologic CH4, but they are not defined anywhere in the manuscript. (b) Then, it is not clear what a ‘thawed glacier bed’ vs ‘frozen glacier bed’ refers to. Since permafrost does not occur beneath most glaciers, presumably thawed vs. frozen refers to the status of the glacier ice at the base of the glacier just above the ground surface. Since the ground beneath the glacier is presumably unfrozen, especially to the depths at which the CH4-rich hydrocarbon layers occur, then groundwater should be able to move through the geologic units irrespective of temperate vs. cold ice at the glacier bed. Therefore, apart from temperate ice being more likely to supply meltwater that can come into contact with geologic CH4, its role in governing CH4 concentrations at the termini of glaciers is not clear. (c) In Fig. 5, the vertical depth scale of the blue arrows under the temperate ice seems arbitrary. I would expect that glacial meltwater permeates more deeply belowground and may resurface anywhere along the glacier bed, including in the cold ice zones. (d) Methane-rich groundwater seepage (indicated in Fig. 5 as a single long blue arrow near the terminus) likely occurs in the cold-ice zone too, implying that it can entrain thermogenic CH4 underlying the cold-ice zone and transport it out of the glacier as recharge to the meltwater river in the low-pressure channel. Thus, more groundwater arrows are needed in the figure, including arrows that demonstrate recharge and discharge beneath the glacier itself.

I am not convinced that glaciers frozen to their bed lack hydrologic contact with geology (as stated on line 214). Even in continuous permafrost regions, where the ground itself can be perennially frozen to depths of hundreds of meters, groundwater springs are in contact with the underlying geology, carrying CH4-rich groundwater to the surface through the otherwise frozen and ice-rich sediments. The sediments beneath glaciers are generally not permafrost, thus it seems even more plausible that the cold-ice portions of glaciers (or at least their low-pressure drainage channel areas) may be ridden with groundwater springs that have been in contact with the coal and shale layers. I would be more convinced of the authors’ idea that temperate ice allows for more entrainment of geologic CH4 and glaciers frozen to their bed lack hydrologic contact with geology if they were able to show that there is no groundwater exchange (e.g. CH4-rich springs) in the cold-ice zone. Can the geophysical data provide insights about subglacial river recharge and discharge points, or at least the dimensions (width and volume) of river channels in the glacial bed so that a sub-glacier water budget could be constructed to provide evidence that groundwater hydrology is complex beneath temperate ice and non-interactive with cold ice zone glacial beds?
Other major comments:

1. Autocorrelation appears to be a problem for the main statistical analysis performed (ANCOVA) to suggest that geology combined with basal temperate ice fraction explains 75% of the variance in terminus meltwater CH4 concentrations. Tto explore the possible correlation between the two explanatory variables I plotted the data provided in Table S1; the two variables appear strongly correlated (see figure below). This autocorrelation violates the assumption of independent variables in ANCOVA. A mixed effects model should be used instead.

2. The geologic data set could be more carefully and thoroughly incorporated into the manuscript's analysis. Geologic units 1-5 were supposedly based on the main geologic unit from a selection of 11 units described in the SI. However, Fig. 4 shows that numerous individual glaciers were underlain by multiple geologic units without a clear dominant unit. Also some units that may have dominated (e.g. Paleocene unit for Glacier 3) are not represented in the 5 categorical units. Faults are known to allow transmission of geologic CH4, but the CH4 data were not related to the fault data.

3. Since another goal of this work was to understand the role of glacial melt on regional CH4 budgets, I would recommend re-analysis of the data to consider the relationships between total glacial river CH4 emissions (not just glacial terminus point CH4 concentrations) and variables such as geology/temperature ice fraction and glacial mass and discharge. The impact of glacial meltwater hydrology clearly extends beyond the terminus of the glacier. Looking at Figure 2, in 11 out of the 19 studied glaciers, CH4 concentrations were lower at the portal site compared to the subsequent 2nd or 3rd downstream samples, suggesting that the primary feed of CH4-rich water to these rivers is groundwater springs beyond the terminus of the glacier. This groundwater likely originates from glacial meltwater in the discharge zone beneath glaciers. The isotopes of water and CH4 could be used to determine how much of the water and CH4 come from glacial melt. As the authors alluded in their Discussion, such an analysis will likely increase the impact of glacial melt on regional CH4 by an order of magnitude.

4. Vertical emissions from the glacier. This paper is concerned with lateral transport of dissolved CH4 out of the meltwater streams; however, other potential pathways of geologic methane release should at least be acknowledged. Ebullition (as opposed to dissolved CH4) releases sub-glacial sourced CH4 near glacial termini. Line 111 suggests that the atmospheric emissions from meltwater streams begin only when water exits the glacial terminus. However, I can imagine that CH4 emissions from subglacial beds may occur prior to the meltwater streams exiting the glacial terminus. Glacial ice has many cracks, moulins and other flowpathays for water and gas. If surface meltwater can find a way down from the surface of glaciers to their beds, then certainly gas can escape upward through these flowpaths to the atmosphere. How do we know that emissions from the glacier itself are not much higher than what is coming out in stream at the terminus, especially if hydrate destabilization is occurring, as is suggested in the Introduction? Have any aerial surveys of CH4 concentrations or fluxes been conducted above these glaciers?

Other comments:

5. The Introduction would benefit from clear statement of hypotheses, providing a compelling scientific argument to capture the interest of the reader.

6. Glaciers are assigned responsibility for both the migration/release (line 47) and sequestration (line 51) of methane. How it is possible for glacial overburden to cause these two seemingly opposing affects needs to be better explained.

7. Lines 61-65. In what way are temperate glaciers a simpler system? Are their beds more uniformly warm, and if so, what is the impact on drainage and on CH4 mobilization and transport?

8. Line 76. Please provide the quantitative values for annual emissions (in Tg CH4 yr-1) associated with permafrost and glaciers in the Svalbard study region, and place this in the context of the total Arctic permafrost budget.

9. Line 79. The previous sentence suggests permafrost and glacial CH4 emissions are comparable in magnitude, but here you say glaciers are the more crucial source. This sounds somewhat contradictory. Why are glaciers more crucial? Do the authors mean to say that compared to permafrost, the potential for glaciers to impact Arctic CH4-climate feedbacks is greater because glacial spatial extent is larger than permafrost soil extent, and the pool of CH4 glaciers cover is greater than that of permafrost soils?

10. Line 86. What is the geographic relationship of the 19 glacial rivers in central Svalbard studied here to the Vallakrabreen catchment, which has been already extensively studied before? Why were results of this study not compared to those of Vallakrabreen?

11. Line 108. Replication. I am surprised about the sample size of n=1 for sampling CH4 concentrations at the glacial termini. Emissions from glacial meltwater streams is known to vary over both diurnal and weekly/monthly time scales. I understand that accessing these sampling locations can be difficult and may not have been feasible to visit sites more than once; however, even on a single visit, triplicate samples at a minimum, would provide a more robust estimate of the subglacial outlet CH4 source for each of the 19 glaciers. What kind of uncertainty does such a small sample size introduce to the interpretation?

12. The Results section is very scarce, and lines 110-112 belong in the Discussion section. I would appreciate to see results reported for 1) downstream trends in CH4 concentration and isotopes; 2) ethane and propane concentrations; 3) dissolved oxygen, electrical conductivity and pH; 4) geophysical surveys, etc. Some of this information is found in the Discussion (it should be in Results), but some is missing entirely in manuscript's analysis (e.g. dissolved O2, pH, EC).

13. Fig. 1. The d13C-Ch4 threshold of -50 ‰ is not a certain indication of thermogenic vs. microbial origin. Microbial CH4 occasionally occurs with values higher than -50 ‰, especially if it has undergone methanotrophy. Typically, other isotopes (dD-CH4, d13C-CO2), other hydrocarbons (ethane, propane, etc), and 14C-CH4 are used in combination with d13C-CH4 to more carefully interpret gas origin. Also, some geologic (fossil) CH4 can have a very light d13C value because it is microbial coalbed CH4; it would be important not to misattribute such a geologic source to microbial decomposition of modern organic matter based on d13C-CH4 alone. Fig S1 is helpful in this regard since it includes the wetness levels together with d13C-CH4; the wetness levels and their interpretation should at least be referenced with Fig. 1, and the manuscript text should include a more nuanced description of isotope geochemical indicators of CH4 origin.

14. Line 135. While I agree that the geology of Svalbard supports the interpretation of a thermogenic origin (as does the combination of heavy d13C and the wetness ratio of the gas), I would like to see a more quantitative consideration of the extent to which methanotrophy may have altered the concentrations and isotope values of the samples. Methanotrophy is a powerful process that reduces emissions to the atmosphere, including potentially the emission of geologic CH4. If it is occurring in glacial terminus (or subglacial stream) environments, it would be good to know. The dissolved oxygen concentrations and redox conditions of all the sampling points should also be provided.

15. Lines 144-148. Svalbard findings are contrasted to Greenland glacial rivers, which have been found to emit microbial CH4 produced from decomposition of young organic matter. Here is an opportunity for an interesting comparison. What are the differences in the magnitudes (not just sources) of emissions for Greenland vs. Svalbard, perhaps normalized to catchment size? I'm curious if the geologic emission are much higher in Svalbard due to the thermogenic CH4 source pool being so much higher?

16. Line 195 indicates that methanotrophy was detected downstream in some river samples via laboratory incubations. Can the authors attempt to estimate the impact of methanotrophy on the CH4 budget of this system? Including an uncertainty analysis will be important.

17. Lines 167-176. Is the interpretation of microbial methanogenesis causing occasional downstream spikes in river CH4 concentrations supported by the wetness ratio too? Please incorporate this dataset in the interpretation and discussion. Also, I am surprised by the suggestion of methanogenesis in the meltwater itself (as opposed to the sediments). Aren't the meltwater streams well oxygenated, especially downstream of the termini?

Minor comments:

Line 87. Change 'accompanied with' to 'accompanied by.'

Line 210. Temperate ice is defined here for the first time, but the definition should be provided much earlier in the manuscript (i.e. at its first use).

Line 294. Sampling was all done downstream of glacier termini, therefore it is not clear why line 294 suggests this downstream sampling point would not include upstream CH4 inputs in the low-pressure channels.

Reviewer #2

(Remarks to the Author)

This is a very good field based study that examines the role of underlying geology and thermal regime on methane sources and fluxes from a variety of different glaciers in Svalbard. The primary conclusion – that methane and associated C2/C3 alkanes fluxes from the glaciers are dominantly thermogenic in origin – appears robust, as does the conclusion that microbial oxidation/methanogenesis in groundwater flows/downstream river sediments are likely altering methane fluxed downstream. I would deem it novel and robust enough to merit consideration for publication in Nature Communications after only minor amendments.

One potential drawback of the study is that the methane measurements at each glacier are based on one time point sampling along spatial transects (from close to portal downstream) in mid summer. However, this approach was pragmatic given the logistical difficulties more comprehensive temporal sampling would involve at such a large number of sample sites, and this caveat (particularly with potential overwinter stored methane) is clearly stated within the text.

Figures are generally well thought through – the design of Fig 1 was excellent, with the different coloured 'bubbles' showing the core results in a concise and easy-to-read manner.

Questions/minor corrections

Main paper

1. Were any replicate samples taken for gas measurements (composition, isotopes) at each site along the transects to allow you to check your overall precision of measurement (i.e. including sampling variance as well as analytical precision).
2. While I presume you have used the differing y scales between glaciers in Fig 2 to enable the reader to see spatial trends, I would suggest plotting the isotopic results on the right hand y axes on the same scales between plots to allow an easier comparison between the different plots.
3. The C2 and C3 data is important data to corroborate a thermogenic origin. To integrate this data better I would be tempted to place Fig 3 in Supplementary and replace it with a figure showing the delta13C-CH4 values versus C1/C2+C2 conc data (with traditional fields of thermogenic vs microbial gases shown), and link to your main text giving more descriptions/analysis of how the 'wetness' varies between different sites.
4. Did you consider taking representative samples of organic-rich rocks from glacier moraines/forefields, crushing them, and analysing their gas evolution/methane isotopic compositions? This could potentially give some further insight into the

primary composition of source gases (and e.g. link into the fig suggested in point 3 above)
5. When discussing the source of gases e.g. in line 322 you write "…..which are predominately thermogenic". I would instead that …."consistent with the delta13C values of thermogenic gases" (and similar throughout text)
6. Abstract – I feel that the line "Our findings offer a completely new way of examining the effect of geology on greenhouse gas release from beneath glaciers" is a little vague/not needed. It reads fine without it.

Supplementary
7. Supplementary Table 1 footnote b) states 'XXX(Louise's method) – please clarify/correct
8. Supplementary Figures 2 and 3; you should define what the shaded error represents (95% c.I.? S.E.?)

Version 2:

Reviewer comments:

Reviewer #1

(Remarks to the Author)
This manuscript presents a valuable data on CH_4 emissions and their origins (mostly thermogenic, some microbial) in meltwater rivers from 19 glaciers with different thermal regimes. The paper also utilizes valuable geophysical data (inferring thermal regimes) and geology (i.e. prevalence of organic-rich shales that are thought to source the thermogenic CH_4) in their analysis. The main goals of the paper are to 1) uncover the mechanism of sub-glacial CH_4 flushing and 2) quantify the magnitude of this source.

Taking the second goal first: The upscaled estimate of CH_4 emissions from glacier outflow rivers presented in this manuscript is ~180 to 380 t CH_4 yr^{-1}, which is an order of magnitude smaller than groundwater springs in the region (Kleber et al. 2023) and about 0.00005% of global atmospheric CH_4 sources. Hence glacial meltwater rivers, even in a shale-rich geologic setting, do not constitute a very big lever in climate feedbacks.

Regarding mechanism, the first goal, I am not convinced that the revised manuscript has adequately uncovered the mechanism of subglacial hydrology in mobilizing and transporting thermogenic CH_4 out from beneath glaciers and to the atmosphere. I suspect that the existing data could be more thoroughly explored to better uncover the mechanism.

Major comments:
(1) I do not agree that glaciers with no temperate ice (i.e. cold-based glaciers) should have little hydrological contact with geology because their beds are frozen First, what pathway does the seasonal meltwater take for cold-ice glaciers? How does it get to the outflow rivers and fjords? Some portion of it must be flowing through the underlying geology. Is it possible that cold-based glaciers simply have less volume of meltwater and therefore make less water available for flushing of subglacial geology? Conversely, does temperate ice indicates more flushing of geologic CH_4 from beneath glaciers simply because there is a larger volume of meltwater in temperate compared to cold-based glaciers?
(2) The original manuscript's Figure 5 attempted to depict the mechanism of meltwater flushing of subglacial geological CH_4. The authors removed this figure; however, such a figure is needed to more clearly communicate how temperate vs. cold ice is thought to regulate meltwater flow pathways in relation to organic-rich shales beneath glaciers.
(3) "Frozen to the bed" indicates to me that the near-surface sediments may be frozen, but it doesn't give any information about how deeply the sediments are frozen or what their hydrologic conductivity through preferential flow paths/taliks may be. If there is permafrost beneath cold-based glaciers it seems probable that glacial meltwater in a cold-based glacier is forced to flow more deeply underground and therefore has a higher likelihood of coming into contact with CH_4-rich shales. Could the authors please add subglacial permafrost to the original manuscript's Figure 5, if they think frozen beds/permafrost plays a role in the system?
(4) Perhaps this CH_4-rich glacial meltwater beneath cold-based glaciers doesn't surface at the portal of the glacier (glacial terminus), but it would surface further downstream, following hydrologic gradients (and supported by downstream data in this manuscript and this team's previous work, Kleber et al. 2023). Therefore, emissions from the full length of the meltwater river systems (normalized to shale abundance and meltwater volume) may be the same for cold-based and temperate ice glaciers. The implication may be that thermal regime doesn't impact total meltwater CH_4 emissions.
To rule out this alternate possibility, the authors could calculate cumulative CH_4 mass for the full length of the river normalized by shale abundance in the underlying geology and total glacial meltwater volume (i.e. total downstream river discharge) to see if there is a significant difference based on temperate ice fraction.
If the alternate scenarios is true (that thermal regime of glacial beds does not impact total emissions from the catchment/full river system), then the major conclusion of this article should be modified to[Line 240]"Our findings indicate that the bed must be thawed and hydrologically active where the glacier meets the shale-rich geology in order for the meltwaters to effectively acquire methane by the time they reach the glacier terminus. Otherwise, for cold-based glaciers, glacial meltwater coming into contact with shale-rich geology, discharges further downstream, but is also ultimately emitted to the atmosphere."

Lines 292-299 support this alternate statement.

The implications of this study may also need to be modified: Future warming should result in accelerated glacial melt with amplified flushing of CH_4 from beneath glaciers AT THE GLACIER TERMINUS when the temperate ice fraction increases, but this increase AT THE GLACIER TERMINUS is likely temporary since transition towards cold-based systems will

eventually reduce the hydrologic connection of meltwater to the glacial terminus stream. However, the downstream hydrology and CH4 emissions implications may need to be further assessed. How much does the dynamic at the glacial terminus impact total geologic emissions (driven by glacial meltwater traveling through groundwater) to the atmosphere along the full length of the river?

Also, it is not clear from the text (line 300, section Potential emissions of methane from glacial rivers) if such a full-river analysis has been done, or if the calculations are only for the CH4 concentrations and discharge at the portal/glacier terminus. Based on Supplementary Table 1, it seems like the analysis refers to the glacial terminus only. The paper would be more meaningful in the context of glacial-CH4-climate feedbacks if a full-meltwater river analysis were conducted.

In lines 56-57, along with the phrase, “Cold-based glaciers have frozen beds, limiting hydraulic conductivity”, please describe the conditions of cold beds in a more quantitative manner. If permafrost sediments are a characteristic of cold beds, please provide the range of depths in which the permanently frozen sediments are known or thought to occur (i.e. permafrost thickness) beneath the Svalbard glaciers, and please add the appropriate references to the manuscript.

In line 58, please provide the range of depths that permanently frozen sediments can occur beneath polythermal glaciers, along with citations. If this information is not known for Svalbard, then mentioning the need for observations and modeling of subglacial permafrost hydrology as a way to improve understanding of glacial meltwater CH4 dynamics would be appropriate in the Discussion. Since strong CH4 emissions are known to occur on Svalbard through intrapermafrost groundwater flow pathways (Hodson 2020), such flowpaths also likely occur beneath glaciers. This implies that glacial meltwater in cold-based glaciers likely finds a way to flow down through frozen sediments and come into contact with shale-rich horizons. Discharge to the river at the portal may not occur under the cold-based glaciers, but likely does occur further downstream. It seems that previous work by these authors (Kleber et al. 2023) supports this concept.

Summary:
-Figure 5 from the previous manuscript should be returned to the paper so that it depicts the authors’ interpretation of subglacial hydrologic connections between meltwater and underlying geology for temperate vs. cold ice. Please include permafrost if/where it is thought to occur, and how these frozen sediments may regulate subglacial hydrologic flow pathways.
-The paper would be more meaningful in the context of glacial-CH4-climate feedbacks if a full-meltwater river analysis were conducted.
- Given that the novelty of glacier meltwater flushing geologic CH4 in this region has already been published by these authors (Kleber et al. 2023), and that this paper is trying to work out the relationship between temperate vs. cold ice in controlling hydrologic connection to the underlying geology, I wonder if this paper would be better suited for a more specialized journal.

Other comments:
Line 75. Please support the statement that CH4-producing soils and wetlands are not expansive and therefore likely a smaller source than glacial catchments by providing quantitative values. The authors’ statement may reflect truth; however, supporting information is needed since hotspots occupy small areas but can dominate emissions. For instance, it would be useful to present the details (area x CH4 flux) for both wetlands and glacial catchments on the archipelago. If all of this information is not available (e.g. because the glacial flux estimate is the goal of this study), then please change the language “are likely’ to “may be” in line 77; but regardless, please provide the areas of each land cover type (organic rich soils/wetlands vs. glacial catchments) and whatever fluxes are already known.

The upscaled estimates of glacial meltwater emissions from this study are provided in line 320. Please provide the estimate for organic-rich soil/wetland emissions as a comparison, along with what is reported for groundwater emissions in lines 339-349.

Line 99. The isotopic composition alone does not indicate CH4 origin. Please change to “the isotopic composition of methane combined with the wetness ratio (C1/(C2+C3)) indicates the likely origin, thermogenic and/or microbial sources.”

Line 105. The abstract says “425 times”; this line says “400 times”. These values are close, but should probably be consistent in the text.

Supplementary Table 1: “bRunoff from ref. XXX (Louise's model)”. This reference still needs to be corrected. Please see Reviewer 2 comments from the previous round.

Reviewer #3

(Remarks to the Author)
This is an field-based study that investigates whether proglacial rivers across Svalbard are a significant source of methane and the factors that control the subglacial methane release. It focuses on 19 glacier catchments where 148 water samples were collected and analysed for methane concentration and carbon isotopic composition to identify methane fluxes and their specific sources. The paper also uses radar - GPR to characterize subglacial thermal regimes and the extent of thawed versus frozen glacier substrates, and is used this to determine how methane concentrations vary with underlying geology, glacier size, and hydrological conditions.

Results reveal that methane supersaturation is widespread in Svalbard's glacial meltwaters, with concentrations attaining up to 400 times atmospheric equilibrium levels. Isotopic and hydrocarbon analyses demonstrate that most of the methane is thermogenic rather than biogenic origin, derived from organic-rich geological formations of shale and coal-bearing rocks rather than primarily from microbial production. The highest methane concentrations occur where glaciers overlie shale-rich bedrock and possessed temperate (i.e. thawed), hydrologically active beds that facilitate active methane transport by subglacial discharge. Although microbial methane production and oxidation occurred in some rivers, these processes were secondary to the dominant geological source.
The authors conclude that glacier meltwaters represent an important pathway for mobilizing ancient (thermogenic) methane stores and that continued glacier retreat will initially enhance methane release through enhanced subglacial hydrological activity. Particularly, the authors speculate that such hydrological conditions might drive substrate hydrofracture, promoting further geological methane flow from shales. However, emissions may eventually decline as glaciers transition to cold-based conditions that limit subglacial meltwater flow and methane transport. The findings highlight a potentially critical climate feedback mechanism and provide a framework for assessing methane emissions from glacierized catchments worldwide.

As far as I am aware of the (considerable) literature on Arctic Svalbard, it is a first of its kind and in my view - will become a benchmark "go to" study. The authors have undertaken a huge amount of work in terms of the field effort to collect the samples, GPR data etc, carried out the hydrocarbon and isotopic analyses, collated these data against the geological controls to arrive at substantive and robust conclusions as to the origin and controls of the CH_4 release across Svalbard. The paper is very well written and presents robust reasoning, justification and arguments. The results and conclusions are important not just locally, but globally for constraining carbon inventories and fluxes. The wider discussion of controls on CH_4 origin and release including glacier thermal regime, subglacial hydrology, geology/lithology is logical and really very well honed. The paper is nicely executed and appropriately pitched for the journal and I foresee that it will be very well cited and, as said, a benchmark for subglacial methane release from this important Arctic region.

I recommend it for publication in Nature Communications with just one minor comment. It has already been iterated through the review process and I would judge that it has benefited in clarity, focus and polish from previous revision(s).

My only comment is that I would like to see a wider and deeper discussion of the methane fluxes (to the atmosphere) here compared with those that have recently been published from rivers emerging along the entire flank of the western Greenland ice sheet (Hatton et al., 2026). The origin of methane from this recently published Greenland study is biogenic and (as pointed out) quite different from the predominantly thermogenic sources identified in this study. Still, it would be useful if the authors could present and evaluate the actual methane fluxes and their relative importance from these contrasting geology/settings and the implication for future subglacial sourced methane release from polar ice sheets given their respective geological settings. Specifically, are the results from this Svalbard (hydrocarbon rich / geothermal) setting significant in terms of the potential geology underlying parts of Greenland and the Antarctic ice sheets where significant hydrocarbon-rich provinces have been mapped/inferred?

Finally, I did briefly look at the author's rebuttal to previous reviews, and feel it is appropriate to comment on what appears to be a misunderstanding from Reviewer 1 regarding the polythermal characterisation (i.e. mixed temperate and cold bedded nature) of Svalbard's glaciers. There is a considerable glaciological literature establishing that many of Svalbard's glaciers are cold-bedded, specifically as they thin and retreat and fail to insulate from a very cold atmospheric signal (see for example, textbooks: Bennett & Glasser, 2009; Benn & Evans, 2010; Caffey & Paterson supplemented by wide-ranging reviews by Irvine-Fynn et al., 2011; etc). My understanding is that the classification "polythermal" glacier was first defined in relation to Svalbard, and it is well established that the ice fabric and beds of glaciers are mixed cold and temperate ice. For this reason, I found Reviewer #1's preoccupation that Svalbard glaciers are temperate bedded quite misled and in my view, the authors are correct to rebut this misconception quite robustly.

References:
Benn, D. and Evans, D. 2010 Glaciers and Glaciation. Second Edition. London, Routledge. https://doi.org/10.4324/9780203785010

Bennett, M. and Glasser, N.F. 2009. Glacial geology: ice sheets and landforms. Second edition. Oxford, Wiley-Blackwell.

Cuffey, K. and Paterson, W.S.B. 2010. The physics of glaciers. Fourth edition. Amsterdam, etc., Academic Press. ISBN-10: 0-123694-61-2.

Hatton, J.E. et al. 2026. Mid-Holocene retreat of the Greenland Ice Sheet indicated by subglacial methane release. Nature Geoscience, pp.1-7.

Irvine-Fynn, T.D. et al., 2011. Polythermal glacier hydrology: A review. Reviews of Geophysics, 49(4).

Version 3:

Reviewer comments:

Reviewer #1

(Remarks to the Author)

This round of revisions and responses to the author has cleared up my misunderstanding and concerns. The critical point that has been resolved is that meltwater for cold-based glaciers runs off at the surface and does not infiltrate into bedrock beneath these glaciers. With this clarification, the interpretation and conclusions make sense and are both interesting and significant. Could the authors add arrows to show this surface runoff for the cold-ice (cold-based portion) of the glacier in Fig. 6? For a broad audience, this would be helpful. I agree with Reviewer 3 that the investigation represents a tremendous amount of work and will be a keystone paper. The comparisons of the thermogenic-CH4 fluxes in Svalbard to those of microbially-dominated CH4 in Greenland makes a strong addition to the revised manuscript.

Reviewer #3

(Remarks to the Author)
I thank the authors for submitting a revised manuscript that adds a paragraph comparing their upscaled (predominantly thermogenic-source) methane results from across Svalbard with up-to-date estimates of that released (biogenic) from the Greenland ice sheet. This small section is neatly written, honed and a useful dimension to the discussion, better placing it within an Arctic - and global - context.

In terms of Reviewer#1's comments - specifically as to whether surface meltwater can access the basal interface of cold / frozen glaciers, the author's have carried out an authoritative and comprehensive rebuttal to this critique. The literature - including major glaciology text books (which they cite) as well as major reviews of polythermal glacier hydrology (also now cited), demonstrate that hydrological systems associated with temperate and cold bedded glaciers are markedly different. The authors' description of cold based glacier regimes - and how it impacts the drainage system - limiting supraglacial meltwater access to the basal environment is quite correct and in-line with all the known literature. Furthermore, their inferences in terms of methane mobilization under contrasting temperate and cold basal conditions are also sound.

My evaluation is that the authors are robust in their analysis and expansive assessment of the mechanisms driving subglacial methane mobilization and release across Svalbard. Given the large inventory of glaciers monitored, along with the excellent geophysical datasets analyzed to support their analysis, this study represents a major contribution to the literature, and will become a "go to" case reference that will no doubt be highly cited over time.

I recommend publication in Nature Communications without further ado.

Open Access This Peer Review File is licensed under a Creative Commons Attribution 4.0 International License, which permits use, sharing, adaptation, distribution and reproduction in any medium or format, as long as you give appropriate credit to the original author(s) and the source, provide a link to the Creative Commons license, and indicate if changes were made.
In cases where reviewers are anonymous, credit should be given to 'Anonymous Referee' and the source.
The images or other third party material in this Peer Review File are included in the article’s Creative Commons license, unless indicated otherwise in a credit line to the material. If material is not included in the article’s Creative Commons license and your intended use is not permitted by statutory regulation or exceeds the permitted use, you will need to obtain permission directly from the copyright holder.

To view a copy of this license, visit https://creativecommons.org/licenses/by/4.0/

Reviewer comments in black.
Author responses in blue.
Changes made to the manuscript in green.

We thank both of the reviewers for their constructive feedback and have provided point-by-point responses to their comments below.

REVIEWER #1

The authors collected CH4 concentration data near the outflows of 19 Svalbard glaciers, plus additional downstream samples along the length of rivers as they flowed away from the glaciers. The region is known for its hydrocarbon-rich lithology, which serves as a source of CH4 in the study rivers. The main idea of this paper is that the temperate ice fraction controls the concentration of CH4 in glacier meltwater streams. The implication is that future warming will increase the temperate ice fraction, mobilizing and releasing to the atmosphere more geologic CH4.

We respectfully clarify that this interpretation does not fully reflect the conclusions presented in the manuscript. Specifically, we do not suggest that future warming will increase the temperate ice fraction. Instead, we state that increased runoff due to accelerated glacier melt may amplify methane flushing from beneath glaciers. This is explicitly stated in the Abstract (lines 29–30): "…suggests that increased runoff due to accelerated glacier melt may amplify methane flushing from beneath glaciers." Furthermore, in the Discussion (lines 261–263), we clarify that methane fluxes from glacier meltwaters are likely to increase temporarily as glaciers continue to melt, but will eventually decline as glaciers transition toward cold-based systems and freeze completely to their beds.

We believe this distinction is critical, as our findings emphasize the transient nature of methane fluxes under future warming scenarios, rather than a sustained increase in methane release due to an increase in temperate ice fraction.

For clarity, my comments will use the meltwater channel as the point of reference when terms 'recharge' and 'discharge' are used. Recharge means that the river is gaining water, while discharge means that it is losing water to the subsurface groundwater reservoir.
My main concerns with the manuscript are insufficient understanding and explanation of sub-glacial hydrology and lack of support for the proposed mechanism of temperate ice fraction control on CH4 concentration.
The interpretation of the data collected is based on a conceptual model (Fig. 5), but details of this model are not described in the text or supported by the observations.

We respectfully disagree with the assertion that the interpretation of the data is based on the conceptual model in Figure 5. Rather, the conceptual model is derived from the interpretation of the data and is intended to summarize and visually communicate the relationships observed in the study. The model is not the basis for the interpretation but rather a synthesis of the findings, which are supported by statistical analyses and observations presented in the manuscript.

For instance, (a) terms such 'high-pressure drainage' and 'low-pressure drainage" appear to be part of the proposed mechanism of meltwater entrainment and transport of geologic CH4, but they are not defined anywhere in the manuscript.

Change to the manuscript: ***We have removed Figure 5, as this conceptual model appears to cause confusion and is perhaps too simple of a representation to accurately show all potential flowpaths of meltwater and groundwater.***

(b) Then, it is not clear what a 'thawed glacier bed' vs 'frozen glacier bed' refers to.

We point out that the distinction between a 'thawed glacier bed' and a 'frozen glacier bed' is explicitly defined in the manuscript. Specifically, lines 221-227 state: "*We use the fraction of temperate ice (i.e., ice at or above its pressure melting point) at the base of the glacier as a proxy for how much of the bed is thawed, and therefore hydrologically active. The presence of temperate ice at the bed of a glacier is strongly indicative of a thawed bed which is hydrologically connected to the drainage system. Glaciers with no temperate ice can be assumed with high certainty to be frozen to their bed and thus there is no hydrological contact with geology*."

This definition provides a clear explanation of the terms and their relevance to the study.

Since permafrost does not occur beneath most glaciers, presumably thawed vs. frozen refers to the status of the glacier ice at the base of the glacier just above the ground surface.

We respectfully disagree with the reviewer's statement that permafrost does not occur beneath most glaciers. The occurrence of permafrost beneath glaciers is well-documented in the scientific literature, particularly in Arctic and sub-Arctic regions such as Svalbard. The following studies are a few examples that provide evidence of permafrost beneath glaciers:

1. **Dobiński et al. (2017): "Cold-temperate transition surface and permafrost base (CTS-PB) as an environmental axis in glacier-permafrost relationship based on research carried out on the Storglaciären and its forefield, northern Sweden" (Quaternary Research).**
2. **Waller et al. (2012): " Glacier–permafrost interactions: Processes, products and glaciological implications" (Sedimentary Geology).**
3. **Fitzsimons et al. (2023): " Subglacial deformation of permafrost beneath Wright Lower Glacier, Antarctica as revealed by direct observations and measurements from a subglacial tunnel" (Geomorphology).**
4. **Reinardy et al. (2019): " Pervasive cold ice within a temperate glacier – implications for glacier thermal regimes, sediment transport and foreland geomorphology" (The Cryosphere).**

In the context of our study, the terms "thawed glacier bed" and "frozen glacier bed" refer to the thermal and hydrological state of the glacier bed, as defined in lines 221-227 of the manuscript. This definition explicitly refers to the thermal state of the glacier bed and its hydrological connectivity to the underlying geology, rather than solely the status of the glacier ice above the ground surface. We believe that our data supports the theory that cold ice indicates a likely frozen bed, thus limiting hydrologic conductivity and in turn, limits the amount of methane that is acquired subglacially. This is why we repeatedly see very low concentrations of methane in cold-based glaciers. We hope this clarification, along with the supporting literature, addresses the reviewer's concern.

Since the ground beneath the glacier is presumably unfrozen, especially to the depths at which the CH4-rich hydrocarbon layers occur, then groundwater should be able to move through the geologic units irrespective of temperate vs. cold ice at the glacier bed.

We disagree with the assumption that the ground beneath the glacier is always unfrozen. As noted in our previous response, the ground beneath glaciers can indeed be frozen, particularly in regions with cold-based ice. The presence of frozen ground

beneath cold-based glaciers thus limits hydrological connectivity between the subglacial hydrological system and the underlying geology.

Regarding the reviewer's point about groundwater movement through geologic units, we agree that groundwater likely flows through the geology beneath the glacier. However, the key mechanism we describe in the manuscript is that *hydraulic conductivity at the glacier bed is restricted in cold-ice zones*. This prevents groundwater or subglacial meltwater from entering the subglacial hydrological system in these zones. In contrast, in temperate ice zones, where the glacier bed is thawed and hydrologically active, groundwater can likely interact with the subglacial drainage system, potentially mobilizing CH4 from the underlying geology.

Change to the manuscript: ***We have added a sentence (lines 250-251) that identifies methane-rich groundwater as a potential source to the subglacial hydrology: "Secondly, methane rich groundwaters may emerge at hydrologically active zones at the glacier bed and join the subglacial drainage system."***

Therefore, apart from temperate ice being more likely to supply meltwater that can come into contact with geologic CH4, its role in governing CH4 concentrations at the termini of glaciers is not clear.

We disagree with the assertion that the role of temperate ice in governing CH4 concentrations at glacier termini is unclear. The presence of temperate ice plays a critical role by enabling hydraulic connectivity between the subglacial hydrological system and the underlying bedrock, allowing meltwater to interact with CH4-rich geologic formations. This mechanism is central to our findings and is supported by our data and the corresponding statistical analyses presented in the manuscript.

Additionally, we describe multiple ways in which thawed areas of the glacier bed (associated with temperate ice) can support methane supersaturation. Specifically, as stated in lines 247-253 of the manuscript:
"Thawed areas of the glacier bed can support methane supersaturation in a variety of ways. First, larger zones of unfrozen glacier bed allow for more water flow, leading to a more extensive subglacial drainage system which can flush out methane stored in the rocks, sediments and cavities below. Secondly, methane rich groundwaters may emerge at hydrologically active zones at the glacier bed and join the subglacial drainage system. Thirdly, water-saturated, anoxic basal sediments create ideal conditions for methanogenesis if any organic substrate is available."

These processes highlight the importance of temperate ice in facilitating both the mobilization of geologic methane and the potential in situ production of methane under anoxic conditions. We believe these explanations sufficiently clarify the role of temperate ice in governing CH4 concentrations at glacier termini.

(c) In Fig. 5, the vertical depth scale of the blue arrows under the temperate ice seems arbitrary. I would expect that glacial meltwater permeates more deeply belowground and may resurface anywhere along the glacier bed, including in the cold ice zones.

We appreciate the reviewer's observations regarding Figure 5. The intention of the figure was to illustrate a simplified conceptual model of the primary pathways through which methane is introduced into the subglacial meltwater system, rather than to depict all potential pathways or the full complexity of subglacial hydrology. However, we see the limitations and therefore potential confusion of the figure and have removed it from the manuscript.

Change to the manuscript: ***We have removed Figure 5, as this conceptual model appears to cause confusion and is perhaps too simple of a representation to accurately show all potential flowpaths of meltwater and groundwater.***

(d) Methane-rich groundwater seepage (indicated in Fig. 5 as a single long blue arrow near the terminus) likely occurs in the cold-ice zone too, implying that it can entrain thermogenic CH_4 underlying the cold-ice zone and transport it out of the glacier as recharge to the meltwater river in the low-pressure channel. Thus, more groundwater arrows are needed in the figure, including arrows that demonstrate recharge and discharge beneath the glacier itself.

(See changes above.)

I am not convinced that glaciers frozen to their bed lack hydrologic contact with geology (as stated on line 214).

We point out that frozen sediments exhibit hydraulic conductivities that are orders of magnitude lower than their unfrozen counterparts, particularly at low temperatures and when ice-saturated. This is well-documented in the literature (ie. Williams and Smith, 1991, Frozen Earth: Fundamentals of Geocryology). As a result, glaciers frozen to their bed are largely hydrologically isolated from the underlying geology.

Even in continuous permafrost regions, where the ground itself can be perennially frozen to depths of hundreds of meters, groundwater springs are in contact with the underlying geology, carrying CH_4-rich groundwater to the surface through the otherwise frozen and ice-rich sediments.

We agree with the reviewer that intra-permafrost groundwater can act as an effective delivery system for subsurface methane, even in regions of continuous permafrost. This is consistent with our observations and is acknowledged in the manuscript. Specifically, lines 281-284 state:
"Intra-permafrost groundwaters still remain effective delivery systems of subsurface methane in these glacial catchments. We see this along nearly all the rivers in this study, where methane concentrations increase abruptly downstream, likely due to groundwater inputs."

This highlights that while frozen sediments beneath cold-based glaciers limit hydrological connectivity at the glacier bed, intra-permafrost groundwater inputs can still contribute to methane concentrations downstream of the glacier terminus. We believe this distinction is important and is already addressed in the manuscript.

The sediments beneath glaciers are generally not permafrost, thus it seems even more plausible that the cold-ice portions of glaciers (or at least their low-pressure drainage channel areas) may be ridden with groundwater springs that have been in contact with the coal and shale layers. I would be more convinced of the authors' idea that temperate ice allows for more entrainment of geologic CH_4 and glaciers frozen to their bed lack hydrologic contact with geology if they were able to show that there is no groundwater exchange (e.g. CH_4-rich springs) in the cold-ice zone.

We respectfully point out that our data provides strong evidence supporting the idea that glaciers frozen to their bed lack significant hydrologic contact with geology. Specifically, glaciers that are entirely or predominantly cold-based exhibit very low methane concentrations in their outflows. This observation suggests that methane-rich groundwater is not entering the subglacial hydrological system in these cold-ice zones. These findings are consistent with the hypothesis that the lack of hydraulic

connectivity in cold-ice zones prevents the entrainment of geologic CH4 into the subglacial meltwater system.

Furthermore, if subglacial groundwaters are flowing into the subglacial hydrological system, it should be reflected by sub-oxic waters emerging at the glacier terminus, as these groundwaters are generally oxygen-depleted. We find that the outflows are consistently fully oxygenated.

While it is challenging to directly measure groundwater exchange in cold-ice zones, the consistently low methane concentrations and completely oxygenated outflows of cold-based glaciers serve as indirect but compelling evidence to support our theory.

Change to the manuscript: ***For reference, we have added dissolved oxygen concentrations of the outflows to the supplementary information.***

Can the geophysical data provide insights about subglacial river recharge and discharge points, or at least the dimensions (width and volume) of river channels in the glacial bed so that a sub-glacier water budget could be constructed to provide evidence that groundwater hydrology is complex beneath temperate ice and non-interactive with cold ice zone glacial beds?

While we do have ground-penetrating radar (GPR) data from some of the glaciers included in this study, the resolution and coverage of these data are not sufficient to resolve the detailed structure of subglacial river channels or to construct a comprehensive subglacial water budget. We use a collection of GPR from different sources. While we have collected some of the data ourselves, these data largely comprise only centrelines of the glaciers. Other GPR data sources include already interpreted profiles, so we do not have access to the raw data. While subglacial conduits can be tracked with dense 3D-processing of GPR, and groundwater springs may theoretically be possible to detect, this would require datasets of much higher resolution than we have access to.

That said, our findings already provide indirect but compelling evidence that subglacial hydrology is more complex and hydrologically active beneath temperate ice zones compared to cold ice zones. Specifically, glaciers with higher temperate ice fractions exhibit significantly higher methane concentrations in their outflows, consistent with the hypothesis that temperate ice facilitates hydraulic connectivity between the subglacial hydrological system and the underlying geology. Conversely, glaciers that are entirely or predominantly cold-based exhibit very low methane concentrations, suggesting limited or no interaction between groundwater and the subglacial hydrological system in these areas.

Other major comments:
1. Autocorrelation appears to be a problem for the main statistical analysis performed (ANCOVA) to suggest that geology combined with basal temperate ice fraction explains 75% of the variance in terminus meltwater CH4 concentrations. Tto explore the possible correlation between the two explanatory variables I plotted the data provided in Table S1; the two variables appear strongly correlated (see figure below). This autocorrelation violates the assumption of independent variables in ANCOVA. A mixed effects model should be used instead.

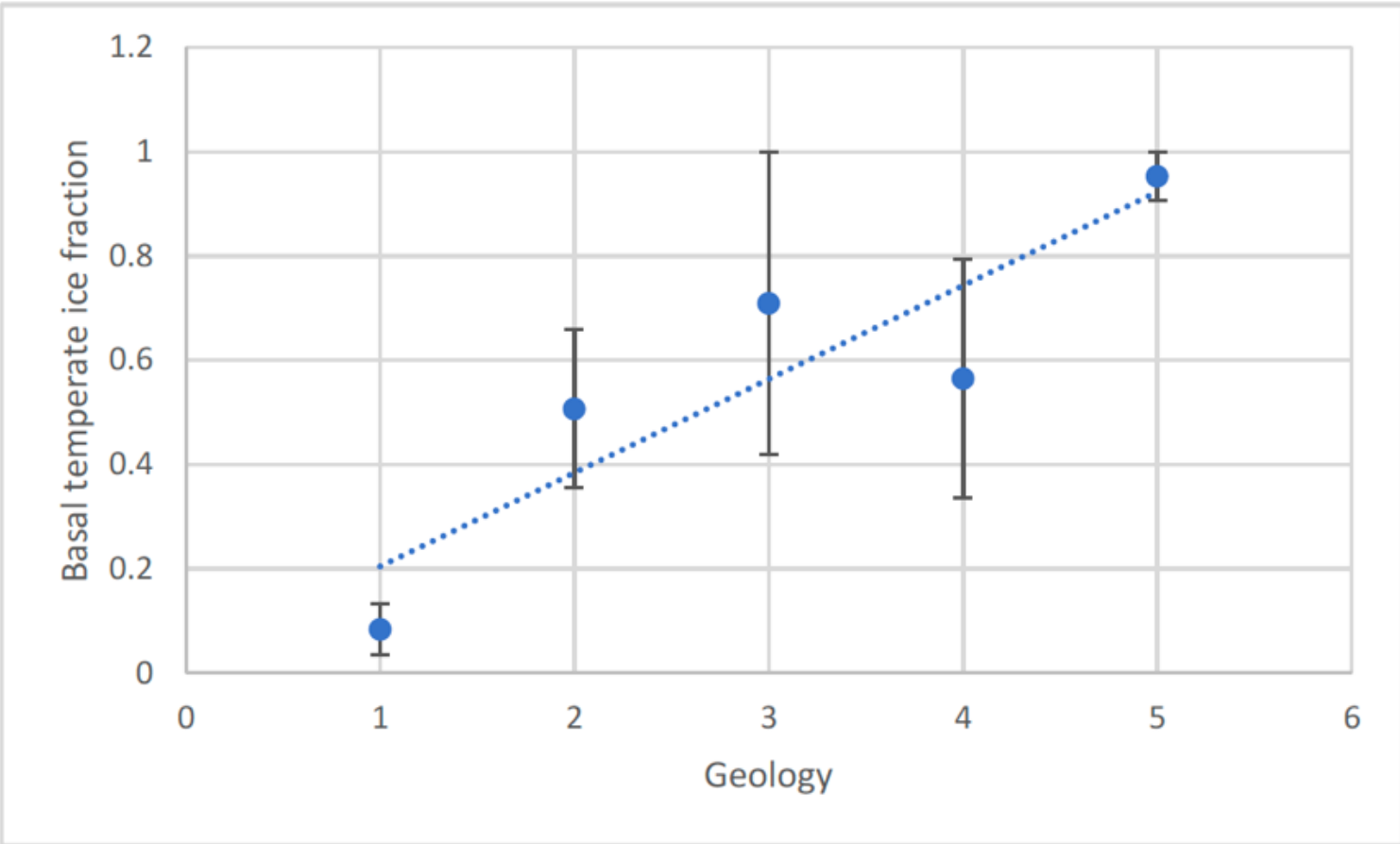

We believe the concern raised pertains to potential *collinearity* between the explanatory variables (bedrock geology and basal temperate ice fraction) rather than *autocorrelation* and will address the concern as such. We appreciate the reviewer bringing this to our attention and have further investigated the potential collinearity between these variables.

ANCOVA assumes that the covariate (basal temperate ice fraction) is independent of the categorical variable (bedrock geology) within the dataset, and that the residuals of the model are normally distributed, homoscedastic, and independent. Importantly, ANCOVA does *not* require that the explanatory variables be completely uncorrelated, but rather that collinearity between them is not excessive. To assess this, we calculated the Variance Inflation Factor (VIF) between bedrock geology and basal temperate ice fraction, which was 4.866. This value is below the conservative threshold of 5 (although the common rule of thumb is a threshold of 10), indicating that collinearity is *not* a significant concern in our model. Additionally, the observed correlation between the two explanatory variables does not inherently violate ANCOVA assumptions, as the residual diagnostics (residual vs. fitted, Q-Q residuals, scale-location, and residuals vs. leverage plots) confirm that the assumptions of the model are met. Also, it is important to note that while the temperate ice fraction and geology show some relation, they share no causality. Geology does not control thermal regime; the apparent relation is random and due to the sample selection.

In our analysis, we considered bedrock geology as the primary factor influencing methane concentrations and first tested its effect using ANOVA. We then added basal temperate ice fraction as a covariate in an ANCOVA model to assess how much additional variation in methane concentrations is explained by basal temperate ice fraction when controlling for geology. This approach allows us to partition the variance in methane concentrations between the two explanatory variables and assess their combined and independent contributions. The residual diagnostics for both the ANOVA and ANCOVA models confirm that the assumptions of normality, homoscedasticity, and independence are met.

To address the reviewer's suggestion, we also tested a mixed-effects model using geology as a fixed effect and basal temperate ice fraction as a continuous covariate. The results were consistent with our ANOVA/ANCOVA analysis. The mixed-effects model explained 75.4% of the variance in log-transformed methane concentrations ($R^2 = 0.754$), with geology and basal temperate ice fraction both contributing significantly to the model ($F = 7.352$, $p = 0.0023$). The coefficient for basal temperate ice fraction was 2.68 ($p = 0.005$), indicating a strong positive relationship with methane concentrations. Among the geology categories, category 2 was associated with

significantly higher methane concentrations compared to the reference category (coefficient = 3.55, p = 0.001). (Results below, BTIF = basal temperate ice fraction.)

	coef	std err	t	P>|t|	[0.025	0.975]
**Intercept**	**0.9995**	**0.991**	**1.009**	**0.333**	**-1.159**	**3.158**
**Geology[T.1]**	**2.3598**	**1.024**	**2.304**	**0.040**	**0.128**	**4.592**
**Geology[T.2]**	**3.5476**	**0.837**	**4.238**	**0.001**	**1.724**	**5.372**
**Geology[T.3]**	**1.9644**	**0.927**	**2.119**	**0.056**	**-0.056**	**3.985**
**Geology[T.4]**	**0.8341**	**0.843**	**0.989**	**0.342**	**-1.003**	**2.672**
**BTIF**	**2.6827**	**0.792**	**3.387**	**0.005**	**0.957**	**4.409**

These results align closely with our ANCOVA findings and support the robustness of our original approach. Given that the mixed-effects model did not provide additional explanatory power or insights beyond the ANCOVA, we have chosen to keep the simpler and more interpretable ANOVA/ANCOVA analysis in the manuscript.

Anyway, we believe the ANOVA/ANCOVA framework is more appropriate for our dataset and research question. Our dataset consists of a single upstream methane concentration measurement per glacier, with no repeated measures or hierarchical structure that would require random effects from a mixed-effects model. Additionally, with our small sample size (n = 18), the ANOVA/ANCOVA avoids overfitting and provides a more parsimonious and interpretable analysis.

2. The geologic data set could be more carefully and thoroughly incorporated into the manuscript's analysis. Geologic units 1-5 were supposedly based on the main geologic unit from a selection of 11 units described in the SI. However, Fig. 4 shows that numerous individual glaciers were underlain by multiple geologic units without a clear dominant unit. Also some units that may have dominated (e.g. Paleocene unit for Glacier 3) are not represented in the 5 categorical units. Faults are known to allow transmission of geologic CH_4, but the CH_4 data were not related to the fault data.

With a total sample size of 19 glacial outlets (18 that we have a basal temperate ice fraction for), it was necessary to minimize the number of geological categories to ensure the statistical robustness of our analysis. We therefore grouped the geologic units into five categories based on well-documented, heavily studied geological groups with similar properties and geological histories.

We acknowledge that some glaciers are underlain by multiple geologic units. In such cases, we selected the geologic unit that accounts for the largest portion of the glacier bed within the transect used for the temperate ice fraction calculation. This approach ensures consistency in how geology is represented in the analysis.

Regarding the Paleocene unit for Glacier 3, we note that it is included within the Eocene geology category and agree that the label in the Figure (which is now Figure 4) should be updated to "Eocene and Paleocene" to more accurately reflect this grouping.

Finally, we acknowledge the reviewer's point about faults potentially influencing CH_4 transmission. While fault data were not explicitly incorporated into the analysis, we agree that faults can play an important role in facilitating the movement of geologic methane. We included faults in Figure 1, but we did not sample from enough glaciers overlying faults to make a concrete statement regarding the effect of faults on our dataset.

Change to the manuscript: ***We have updated the figure (now Fig. 4) to include Paleocene in the label.***

3. Since another goal of this work was to understand the role of glacial melt on regional CH4 budgets, I would recommend re-analysis of the data to consider the relationships between total glacial river CH4 emissions (not just glacial terminus point CH4 concentrations) and variables such as geology/temperature ice fraction and glacial mass and discharge. The impact of glacial meltwater hydrology clearly extends beyond the terminus of the glacier. Looking at Figure 2, in 11 out of the 19 studied glaciers, CH4 concentrations were lower at the portal site compared to the subsequent 2nd or 3rd downstream samples, suggesting that the primary feed of CH4-rich water to these rivers is groundwater springs beyond the terminus of the glacier. This groundwater likely originates from glacial meltwater in the discharge zone beneath glaciers. The isotopes of water and CH4 could be used to determine how much of the water and CH4 come from glacial melt. As the authors alluded in their Discussion, such an analysis will likely increase the impact of glacial melt on regional CH4 by an order of magnitude.

The primary goal of this study was to determine the factors controlling the mobilization of methane within the subglacial hydrology and how these factors influence the methane transported from beneath the glacier and out from the glacier terminus. For this reason, we focused on the most upstream sampling point, closest to the glacier terminus, to conduct this portion of the analysis.

We fully agree with the reviewer that the impact of glacial meltwater hydrology extends beyond the terminus of the glacier, which is why we collected transects of samples along the length of each river to measure methane concentrations downstream. This allowed us to observe that additional methane inputs often occur in the proglacial areas. As noted in the manuscript (lines 281-287):
"Intra-permafrost groundwaters still remain effective delivery systems of subsurface methane in these glacial catchments. We see this along nearly all the rivers in this study, where methane concentrations increase abruptly downstream, likely due to groundwater inputs. In fact, peak methane concentrations are reached far downstream from the glacier terminus in 68% of the rivers in this study, indicating that the methane flux from groundwaters is potentially more critical than the methane delivered by the subglacial drainage system."

Additionally, in lines 335-338, we highlight the broader implications of this finding:
"Our findings suggest that glacial meltwaters are not the most critical natural methane source on Svalbard. Rather, emissions from terrestrial seepage points of methane-rich groundwaters such as those found in glacial forefields and pingos appear to be more important for the Arctic methane budget."

It is important to note that our previous work (Kleber et al., 2023, *Nature Geoscience*, https://www.nature.com/articles/s41561-023-01210-6) has already demonstrated the importance of glacial groundwaters in contributing to methane emissions across Svalbard. This study builds on that foundation by focusing specifically on the methane emissions from meltwaters emerging directly from beneath glaciers, which would not have been incorporated into the wider groundwater methane flux calculations presented in our earlier work. By isolating the subglacial meltwater contribution, we aim to better understand the processes governing methane mobilization within the subglacial hydrology itself.

Regarding the suggestion to use isotopic data of water and CH4 to determine the contributions of glacial meltwater and groundwater, we agree that this would provide valuable insights. However, isotopic data were not collected as part of this study, so

this type of analysis is not possible with the current dataset, and in light of our comments above, is beyond the scope of our study.

4. Vertical emissions from the glacier. This paper is concerned with lateral transport of dissolved CH4 out of the meltwater streams; however, other potential pathways of geologic methane release should at least be acknowledged. Ebullition (as opposed to dissolved CH4) releases sub-glacial sourced CH4 near glacial termini. Line 111 suggests that the atmospheric emissions from meltwater streams begin only when water exits the glacial terminus. However, I can imagine that CH4 emissions from subglacial beds may occur prior to the meltwater streams exiting the glacial terminus. Glacial ice has many cracks, moulins and other flowpathays for water and gas. If surface meltwater can find a way down from the surface of glaciers to their beds, then certainly gas can escape upward through these flowpaths to the atmosphere. How do we know that emissions from the glacier itself are not much higher than what is coming out in stream at the terminus, especially if hydrate destabilization is occurring, as is suggested in the Introduction? Have any aerial surveys of CH4 concentrations or fluxes been conducted above these glaciers?

We appreciate the reviewer's comments regarding potential vertical methane emissions from glaciers and acknowledge that this is an important topic for understanding the full methane budget of glacial systems. However, the primary focus of our study was to investigate the *lateral transport of methane by meltwater streams emerging from beneath glaciers*. While vertical emissions from moulins, crevasses, or other pathways may occur, this was beyond the scope of our study, which specifically aimed to quantify methane mobilization and transport within the subglacial hydrological system and its subsequent release via glacial meltwater streams.

Regarding the reviewer's interpretation of line 111 ("In glacial rivers where water temperatures are near 0°C, excess methane above ~4 nM is out of equilibrium with the atmosphere and begins to diffuse, or degas, from the rivers as they flow downstream..."), we emphasize that this statement does not discount the possibility of methane emissions occurring prior to the water exiting the glacier terminus. In fact, we address this in the manuscript (lines 310-313), where we state:
"Our methane emission projections are likely conservative because they are based on our most upstream sampling points and therefore do not account for methane degassing upstream of the termini in low-pressure channels, or between the termini and the sampling points."

We agree with the reviewer that investigating methane emissions from moulins, crevasses, or other vertical pathways would be an interesting and valuable area of research. However, such an analysis would require a completely different methodological approach and is not typically included in studies focused on glacial river methane emissions. We suggest this as an important direction for future research, as it would complement our findings and provide a more comprehensive understanding of methane emissions from glacial systems.

Other comments:
5. The Introduction would benefit from clear statement of hypotheses, providing a compelling scientific argument to capture the interest of the reader.

We believe that the study objectives are clearly stated in the last paragraph of the introduction and that explicitly stating our hypotheses is not necessary and would not improve the introduction of the manuscript. This is also not standard in other *Nature Communications* publications.

6. Glaciers are assigned responsibility for both the migration/release (line 47) and sequestration (line 51) of methane. How it is possible for glacial overburden to cause these two seemingly opposing affects needs to be better explained.

We respectfully point out that the second and third paragraphs of the Introduction explicitly describe how glaciers can both sequester methane and facilitate its release. This section clearly outlines the dual role of glaciers in both sequestering and releasing methane, depending on the specific processes and conditions at play.

7. Lines 61-65. In what way are temperate glaciers a simpler system? Are their beds more uniformly warm, and if so, what is the impact on drainage and on CH4 mobilization and transport?

The statement in lines 61–65 is intended to highlight that the hydrology of polythermal glaciers is complicated by the presence of both cold and temperate ice at their beds. This thermal heterogeneity significantly influences the configuration of the subglacial drainage system, as cold ice zones are frozen to the bed and lack hydrological connectivity, while temperate ice zones are thawed and hydrologically active.
The reference we cite for these lines (Irvine-Fynn et al., 2011, *Reviews of Geophysics*, https://doi.org/10.1029/2010RG000350) provides a detailed discussion of the diversity and complexity of polythermal glacier thermal structures and their implications for subglacial hydrology. It explains how the thermal structure of a glacier influences the extent and configuration of its drainage system, with polythermal glaciers exhibiting a more spatially variable and discontinuous hydrological network compared to the more uniformly warm beds of temperate glaciers.

The hydrology of temperate glaciers, where the bed is uniformly warm and thawed, allows for a more continuous and extensive subglacial drainage system. This uniformity likely facilitates more consistent mobilization and transport of CH4 from the subglacial environment. In contrast, the thermal heterogeneity of polythermal glaciers creates a more fragmented drainage system, which may limit or compartmentalize CH4 mobilization and transport in certain areas.

Change to the manuscript: ***We have changed the words "more complex" in these lines to "more spatially variable" to make this point clearer.***

8. Line 76. Please provide the quantitative values for annual emissions (in Tg CH4 yr-1) associated with permafrost and glaciers in the Svalbard study region, and place this in the context of the total Arctic permafrost budget.

No quantitative estimates currently exist for methane emissions from permafrost thaw on Svalbard. This is due to the scarcity of measurements and the spatial heterogeneity of the available data, as noted in the manuscript.

Similarly, no upscaled quantitative value currently exists for methane emissions from glacial meltwaters on Svalbard, which is precisely the knowledge gap that our study aims to address. By focusing on methane mobilization and transport in subglacial hydrology, we aim to provide the first estimates of upscaled methane emissions from glacial meltwaters in this region.

Quantitative estimates do exist for methane emissions from glacial groundwaters on Svalbard, which were reported in our previous work (Kleber et al., 2023, *Nature Geoscience*). These emissions are estimated to be up to 2.31 kt CH_4 per year, and this value is included in the discussion section of the manuscript (lines 306–317).

Change to the manuscript: ***We made this more explicit in the text (lines 332-339): "The amount of methane mobilized from beneath glaciers can be compared to other natural methane emission sources on Svalbard, such as groundwater and thawing permafrost. Our calculated ranges are approximately an order of magnitude lower than the projected annual methane emissions from glacial groundwater across Svalbard, which are estimated to be up to 2.31 kt CH4 per year. In contrast, upscaled methane yield estimates from Svalbard permafrost remain unavailable due to a scarcity of measurements and the spatial variability of existing data. The limited studies conducted report highly variable localized fluxes, with one even suggesting that the Svalbard tundra can act as a net sink for methane."***

We also added a statement about the only glacial river that has been studied previously on Svalbard in terms of methane emissions. See Point 10 below.

9. Line 79. The previous sentence suggests permafrost and glacial CH4 emissions are comparable in magnitude, but here you say glaciers are the more crucial source. This sounds somewhat contradictory. Why are glaciers more crucial? Do the authors mean to say that compared to permafrost, the potential for glaciers to impact Arctic CH4-climate feedbacks is greater because glacial spatial extent is larger than permafrost soil extent, and the pool of CH4 glaciers cover is greater than that of permafrost soils?

We appreciate the reviewer's observation and clarify that the statement refers specifically to glacial catchments as a more crucial methane source on Svalbard, not glaciers themselves. This is due to two key factors: (1) organic-rich soils and wetlands, the primary sources of permafrost methane emissions, are limited on Svalbard, and (2) glacial catchments release significant methane-rich groundwater, as shown in our previous work (Kleber et al., 2023, *Nature Geoscience*).

Change to the manuscript: ***We have updated the sentence to clarify: "While permafrost thaw on Svalbard can result in high methane emissions per unit area, the organic-rich soils and wetlands that yield high rates of methane production are not expansive on the archipelago. Consequently, methane emissions from Svalbard's extensive glacial catchments are likely a more significant source of methane to the atmosphere."***

10. Line 86. What is the geographic relationship of the 19 glacial rivers in central Svalbard studied here to the Vallakrabreen catchment, which has been already extensively studied before? Why were results of this study not compared to those of Vallakrabreen?

Vallåkrabreen is one of the 19 glaciers included in this study. Our findings from previous work on Vallåkrabreen (*Kleber et al., 2025, Biogeosciences*, https://bg.copernicus.org/articles/22/659/2025/) yielded annual methane emission estimates that are highly consistent with those of the current study.

Change to the manuscript: ***We have included a statement comparing our findings to the previous study on Vallåkrabreen in lines 298-302: "Only one previous study has investigated methane export from glacial melt rivers on Svalbard, where 618 kg CH4 (486-768 kg) were found to be transported from beneath the Vallåkrabreen glacier across the 2021 melt season. Our estimated methane export from beneath Vallåkrabreen (16), which is up to 607 kg CH4 a-1 (580-632 kg CH4 a-1), is highly consistent with these previous findings."***

11. Line 108. Replication. I am surprised about the sample size of n=1 for sampling CH4 concentrations at the glacial termini. Emissions from glacial meltwater streams is known to vary over both diurnal and weekly/monthly time scales. I understand that accessing these sampling locations can be difficult and may not have been feasible to visit sites more than once; however, even on a single visit, triplicate samples at a minimum, would provide a more robust estimate of the subglacial outlet CH4 source for each of the 19 glaciers. What kind of uncertainty does such a small sample size introduce to the interpretation?

To clarify, two to three replicate samples were taken at each sampling point, and the reported concentrations represent the average of these replicate measurements.

Change to the manuscript: ***We have updated the methods section (lines 360-361) to clarify this.***

We also recognize that methane emissions from glacial meltwater streams can vary diurnally and seasonally. This is acknowledged in the manuscript (lines 313-316): "Our sampling was conducted during mid-summer and therefore does not capture the pulse of methane that has been stored beneath glaciers during winter and is flushed out at the onset of the melt season. This can be a substantial portion of the total melt season methane flux."

As the reviewer notes, accessing these remote sampling locations was logistically challenging, and repeat visits to the same sites were not feasible. We acknowledge that sampling at a single time point introduces some uncertainty into the interpretation, as temporal variability is not captured. However, this limitation is unavoidable given the logistical constraints of fieldwork in such remote environments. We believe that the large number of sites sampled (19 glacier sites, which effectively doubles the total number of sites with published data of this kind worldwide) provides a strong basis for the conclusions drawn in this study, and we have addressed this limitation in the manuscript.

12. The Results section is very scarce, and lines 110-112 belong in the Discussion section. I would appreciate to see results reported for 1) downstream trends in CH4 concentration and isotopes; 2) ethane and propane concentrations; 3) dissolved oxygen, electrical conductivity and pH; 4) geophysical surveys, etc. Some of this information is found in the Discussion (it should be in Results), but some is missing entirely in manuscript's analysis (e.g. dissolved O2, pH, EC).

We appreciate the reviewer's feedback regarding the structure and content of the Results section. The current structure was designed as a combined Results and Discussion section, which aligns with typical formats of many Nature Portfolio journals, including research articles published in *Nature Communications*. As a short-form article, we believe that providing all the data requested in the main text would make the article too lengthy. We have provided all river data in the Supplementary Information. However, the geophysical surveys are published in a separate publication, which is referenced in the methods text.

Change to the manuscript: ***We have added the dissolved oxygen, electrical conductivity and pH data, as available, in Supplementary Table 2. A citation has been added to the methods section to the publication in which the data from the geophysical surveys has been published.***

13. Fig. 1. The d13C-Ch4 threshold of -50 ‰ is not a certain indication of thermogenic vs. microbial origin. Microbial CH4 occasionally occurs with values higher than -50 ‰, especially if it has undergone methanotrophy. Typically, other isotopes (dD-CH4, d13C-CO2), other

hydrocarbons (ethane, propane, etc), and 14C-CH4 are used in combination with d13C-CH4 to more carefully interpret gas origin. Also, some geologic (fossil) CH4 can have a very light d13C value because it is microbial coalbed CH4; it would be important not to misattribute such a geologic source to microbial decomposition of modern organic matter based on d13C-CH4 alone. Fig S1 is helpful in this regard since it includes the wetness levels together with d13C-CH4; the wetness levels and their interpretation should at least be referenced with Fig. 1, and the manuscript text should include a more nuanced description of isotope geochemical indicators of CH4 origin.

We agree that $\delta^{13}C$-CH_4 alone is not a definitive indicator of thermogenic vs. microbial methane origin. In the manuscript, we use the $\delta^{13}C$-CH_4 values as a general guide, with color coding in the figures to help readers quickly interpret where each measurement lies on the spectrum, rather than to assign a definitive origin. We also acknowledge in the text that isotopic composition can be altered by methanotrophy (lines 131–138):
"It is important to consider that the isotopic composition of methane can be altered by the microbial oxidation of some of that methane, a process known as methanotrophy. Methanotrophy causes the isotopic composition of the remaining methane to become less negative, moving towards the thermogenic range. However, we suspect that the thermogenic signatures we observe are not an effect of subglacial methane oxidation, but rather due to the abundant sources of thermogenic methane found on Svalbard."

To strengthen the interpretation, we also include measurements of other hydrocarbons (ethane and propane) and calculate wetness levels to provide additional evidence of a largely thermogenic source.

Change to the manuscript: ***We have added the ethane and propane data to the main text by moving the 'wetness' figure from the supplementary into the main text, which is now Figure 2, along with a discussion in lines 135-144.***

14. Line 135. While I agree that the geology of Svalbard supports the interpretation of a thermogenic origin (as does the combination of heavy d13C and the wetness ratio of the gas), I would like to see a more quantitative consideration of the extent to which methanotrophy may have altered the concentrations and isotope values of the samples. Methanotrophy is a powerful process that reduces emissions to the atmosphere, including potentially the emission of geologic CH4. If it is occurring in glacial terminus (or subglacial stream) environments, it would be good to know. The dissolved oxygen concentrations and redox conditions of all the sampling points should also be provided.

We agree that subglacial methanotrophy could potentially alter methane concentrations and isotopic values at the glacier terminus. However, the primary focus of this study is to calculate the *net methane transport from the glacier bed* via subglacial hydrology and suggest the source of the methane, rather than to quantify the extent of methanotrophy or the total methane produced and consumed within the subglacial environment. A quantitative assessment of the extent of methanotrophy would require a much more extensive dataset, which is beyond the scope of this study.

We appreciate the reviewer's suggestion and agree that further investigation into subglacial methanotrophy would be valuable and we suggest this as an important direction for future research.

Change to the manuscript: ***We have added the dissolved oxygen concentrations to Supplementary Table 2. We have also enhanced our discussion on methanotrophy in***

lines 130-133 to state that methanotrophy has been found to occur in the subglacial environment on Greenland.

In our new Figure 2, we have included a line to indicate the direction that oxidation (or methanotrophy) would shift the wetness levels and the isotopes of methane on the plot. If extensive methanotrophy was occurring, one would expect to see a skew in the data accordingly. We have added a line that addresses this in the manuscript (lines 132-135): "Methanotrophy enriches the remaining methane in 13C, causing its isotopic signature to shift towards the thermogenic range, as indicated by the 'oxidation' arrow in Fig. 2. Our data does not display a general skew in this direction, as would be expected if the samples represented various extents of methane oxidation."

15. Lines 144-148. Svalbard findings are contrasted to Greenland glacial rivers, which have been found to emit microbial CH4 produced from decomposition of young organic matter. Here is an opportunity for an interesting comparison. What are the differences in the magnitudes (not just sources) of emissions for Greenland vs. Svalbard, perhaps normalized to catchment size? I'm curious if the geologic emission are much higher in Svalbard due to the thermogenic CH4 source pool being so much higher?

Very few studies exist that quantify total methane fluxes from meltwaters exiting glaciers, making an extensive comparison difficult.

Change to the manuscript: ***We have added to the text of this discussion in lines 160-167: "A previous study of the Vallåkrabreen glacier on Svalbard found that up to six-times more methane per glacier area is transported from the subglacial environment by its meltwaters during the melt season than is transported by the meltwaters of Leverett Glacier in Greenland, which contain microbially produced methane. The study suggests that the subglacial waters of Vallåkrabreen flush geologic methane from fractures in the organic-rich shale below, leading to greater methane mobilization."***

16. Line 195 indicates that methanotrophy was detected downstream in some river samples via laboratory incubations. Can the authors attempt to estimate the impact of methanotrophy on the CH4 budget of this system? Including an uncertainty analysis will be important.

We agree that estimating the impact of methanotrophy on the CH_4 budget would be valuable; however, this is extremely challenging with the current dataset. Furthermore, our laboratory incubations showed net *generation* of methane in river samples, and only showed net consumption of methane (or methanotrophy) in groundwater samples. Nevertheless, our isotopic data show that there are multiple sources (with varying isotopic compositions) and sinks of methane along the rivers, making it impossible to use isotopic and concentration changes to calculate rates of methanotrophy. Furthermore, these sources and sinks, as well as their rates, vary significantly among the different glacial rivers, adding further complexity.

Additionally, this analysis is outside the scope of the current study, which focuses on estimating the amount of methane transported from the glacier bed and the potential emissions from meltwater streams. Our goal is to quantify the methane emerging from the glacier terminus, not to calculate net emissions after accounting for downstream processes such as methanotrophy.

Change to the manuscript: ***We have updated our discussion of methanotrophy in lines 318-326: "However, our isotopic measurements indicate that some methane may be***

oxidized in-stream in some of our catchments and thus not all is released to the atmosphere. It is not possible to quantify the extent of oxidation with our available data because instream microbial production of methane and inputs of methane-rich groundwater occur between sampling points, thus further altering the concentrations and isotopic compositions of the methane. While methane oxidation rates within rivers vary widely among studies, it is generally found that the rate of methane outgassing from a river is much faster than the rate of microbial oxidation, especially in shallow rivers with high surface-area-to-volume ratios, like the rivers in this study."

17. Lines 167-176. Is the interpretation of microbial methanogenesis causing occasional downstream spikes in river CH4 concentrations supported by the wetness ratio too? Please incorporate this dataset in the interpretation and discussion. Also, I am surprised by the suggestion of methanogenesis in the meltwater itself (as opposed to the sediments). Aren't the meltwater streams well oxygenated, especially downstream of the termini?

We would like to clarify that we do not attribute the downstream spikes in CH_4 concentrations to methanogenesis. As stated in the manuscript (lines 173-178), these spikes are most likely caused by inputs of methane-rich groundwater:
"We also observe frequent additional recharge of methane along the river flowpaths. Abrupt increases in methane concentration downstream from the glacier terminus, as observed in the Gløttfjellebreen (2) and Vestre Grønfjordbreen (5) rivers, are likely due to discrete sources of methane-rich groundwater flowing into the river, which are common in glacier forefields."

We do, however, suggest that isotopic changes along some rivers indicate instream microbial methanogenesis occurring in riverbed *sediments*, which could be anoxic and conducive to methanogenesis. We do not suggest that methanogenesis is occurring in the oxygenated meltwater itself. This distinction is stated in lines 178-180: "Furthermore, isotopic changes along some of the rivers suggest that while methane is outgassing from the river, it is continually supplemented by the instream microbial production of methane, or methanogenesis, in riverbed sediments." We also show that methane has been produced during incubation experiments of river water (lines 193-195).

Regarding the wetness ratio, we observe some changes in the wetness ratio that coincide with isotopic changes suggesting methanogenesis. However, the number of data points is limited, and we cannot confidently use this as definitive evidence.

Change to the manuscript: ***We have incorporated the wetness data in the manuscript in our new Figure 2.***

Minor comments:
Line 87. Change 'accompanied with' to 'accompanied by.'

Change to the manuscript: ***We have updated the manuscript accordingly.***

Line 210. Temperate ice is defined here for the first time, but the definition should be provided much earlier in the manuscript (i.e. at its first use).

Change to the manuscript: ***We have provided the definition of temperate ice at its first mention (lines 54-56): "The temperature and hydrological conditions of the subglacial environment are governed by the glacier's thermal conditions, or the extent of 'cold' (sub-freezing) versus temperate ('warm,' above the pressure melting point) ice."***

Line 294. Sampling was all done downstream of glacier termini, therefore it is not clear why line 294 suggests this downstream sampling point would not include upstream CH_4 inputs in the low-pressure channels.

The statement referred to by the reviewer ("*These projections are likely conservative because they are based on our most upstream sampling points and therefore do not account for methane degassing upstream of the termini in low pressure channels, or between the termini and the sampling points."*) does not discuss any CH_4 *inputs*. Rather, it is stating that any CH_4 *lost* (specifically, "degassed") upstream of the sampling point would not be considered in our analyses.

REVIEWER #2

This is a very good field based study that examines the role of underlying geology and thermal regime on methane sources and fluxes from a variety of different glaciers in Svalbard. The primary conclusion – that methane and associated C2/C3 alkanes fluxes from the glaciers are dominantly thermogenic in origin – appears robust, as does the conclusion that microbial oxidation/methanogenesis in groundwater flows/downstream river sediments are likely altering methane fluxed downstream. I would deem it novel and robust enough to merit consideration for publication in Nature Communications after only minor amendments.

One potential drawback of the study is that the methane measurements at each glacier are based on one time point sampling along spatial transects (from close to portal downstream) in mid summer. However, this approach was pragmatic given the logistical difficulties more comprehensive temporal sampling would involve at such a large number of sample sites, and this caveat (particularly with potential overwinter stored methane) is clearly stated within the text.

We thank the reviewer for their helpful comments and improvements to our manuscript. We now see that our sampling replication was not clearly stated in the manuscript, however two to three replicate samples were taken at each sampling point, and the reported concentrations represent the average of these replicate measurements.

Change to the manuscript: ***We have updated the methods section (lines 360-361) to clarify this.***

Figures are generally well thought through – the design of Fig 1 was excellent, with the different coloured 'bubbles' showing the core results in a concise and easy-to-read manner.

Questions/minor corrections

Main paper

1. Were any replicate samples taken for gas measurements (composition, isotopes) at each site along the transects to allow you to check your overall precision of measurement (i.e. including sampling variance as well as analytical precision).

(See above.)

2. While I presume you have used the differing y scales between glaciers in Fig 2 to enable the reader to see spatial trends, I would suggest plotting the isotopic results on the right hand y axes on the same scales between plots to allow an easier comparison between the different plots.

Change to the manuscript: ***We agree that a standardized scale for the isotopic results is much clearer and have updated the figure accordingly (which is now Fig. 3).***

3. The C2 and C3 data is important data to corroborate a thermogenic origin. To integrate this data better I would be tempted to place Fig 3 in Supplementary and replace it with a figure showing the delta13C-CH4 values versus C1/C2+C2 conc data (with traditional fields of thermogenic vs microbial gases shown), and link to your main text giving more descriptions/analysis of how the 'wetness' varies between different sites.

Change to the manuscript: ***We agree that the C2 and C3 data is important and have moved the 'wetness' figure from the supplementary into the main text, which is now Figure 2.***

4. Did you consider taking representative samples of organic-rich rocks from glacier moraines/forefields, crushing them, and analysing their gas evolution/methane isotopic compositions? This could potentially give some further insight into the primary composition of source gases (and e.g. link into the fig suggested in point 3 above)

We agree that this is a great idea that would provide valuable insights, however we did not manage to take such samples. Such studies have been conducted previously on Svalbard and we have added this to the discussion.

Change to the manuscript: ***Lines 141-150: "Ethane and propane concentrations measured in our river samples yield wetness (C1/(C2+C3)) levels ranging from 3.7 to 91.4 (n = 61). When coupled with the carbon isotopic signatures, these wetness levels indicate that the methane originates from oil-associated, early mature or late mature thermogenic gas (Fig. 2). The skew of our data in the direction of the 'migration' arrow in Fig. 2 also suggests that the methane in our samples have been captured at various stages of migration away from source rocks. These findings are consistent with previous geochemical observations of gaseous hydrocarbons in similar geological settings on Svalbard, where largely thermogenic gases at varying degrees of maturity are found to have migrated from several primary source rocks."***

5. When discussing the source of gases e.g. in line 322 you write "…..which are predominately thermogenic". I would instead that ….”consistent with the delta13C values of thermogenic gases" (and similar throughout text)

Change to the manuscript: ***We have updated the text to more clearly connect the identified source with the isotopic signature. For example, the above-mentioned line has been changed to: "Glaciers situated on shale-rich geological formations with thawed, hydrologically active beds mobilize significantly greater methane fluxes, largely of thermogenic origin, as corroborated by isotopic signatures and wetness levels of dissolved gases."***

6. Abstract – I feel that the line "Our findings offer a completely new way of examining the effect of geology on greenhouse gas release from beneath glaciers" is a little vague/not needed. It reads fine without it.

Change to the manuscript: ***We have removed the sentence.***

Supplementary

7. Supplementary Table 1 footnote b) states 'XXX(Louise's method) – please clarify/correct

Change to the manuscript: ***Thank you for catching this! We have corrected the error.***

8. Supplementary Figures 2 and 3; you should define what the shaded error represents (95% c.I.? S.E.?)

Change to the manuscript: ***We have added to the caption of each figure that the shaded error represents the standard error of the mean.***

Reviewer comments in black.
Author responses in blue.
Changes to the manuscript in red.

Reviewer #1 (Remarks to the Author):

This manuscript presents a valuable data on CH4 emissions and their origins (mostly thermogenic, some microbial) in meltwater rivers from 19 glaciers with different thermal regimes. The paper also utilizes valuable geophysical data (inferring thermal regimes) and geology (i.e. prevalence of organic-rich shales that are thought to source the thermogenic CH4) in their analysis. The main goals of the paper are to 1) uncover the mechanism of sub-glacial CH4 flushing and 2) quantify the magnitude of this source.

Taking the second goal first: The upscaled estimate of CH4 emissions from glacier outflow rivers presented in this manuscript is ~180 to 380 t CH4 yr-1, which is an order of magnitude smaller than groundwater springs in the region (Kleber et al. 2023) and about 0.00005% of global atmospheric CH4 sources. Hence glacial meltwater rivers, even in a shale-rich geologic setting, do not constitute a very big lever in climate feedbacks.

Regarding mechanism, the first goal, I am not convinced that the revised manuscript has adequately uncovered the mechanism of subglacial hydrology in mobilizing and transporting thermogenic CH4 out from beneath glaciers and to the atmosphere. I suspect that the existing data could be more thoroughly explored to better uncover the mechanism.

Major comments:
(1) I do not agree that glaciers with no temperate ice (i.e. cold-based glaciers) should have little hydrological contact with geology because their beds are frozen First, what pathway does the seasonal meltwater take for cold-ice glaciers? How does it get to the outflow rivers and fjords? Some portion of it must be flowing through the underlying geology. Is it possible that cold-based glaciers simply have less volume of meltwater and therefore make less water available for flushing of subglacial geology? Conversely, does temperate ice indicates more flushing of geologic CH4 from beneath glaciers simply because there is a larger volume of meltwater in temperate compared to cold-based glaciers?

We thank Reviewer #1 for their time and consideration of our manuscript and their helpful comments and perspectives. We will address their above questions individually:

What pathway does the seasonal meltwater take for cold-ice glaciers? Meltwater from cold-based glaciers is largely limited to surface runoff. They tend to be undynamic systems which do not form crevasses and therefore lack a pathway for meltwater to reach the bed.
How does it get to the outflow rivers and fjords? The meltwater flows in channels on the glacier surface, which flow into the lateral or proglacial area and typically join to make up the main glacial melt river, which eventually flows to the fjord.
Some portion of it must be flowing through the underlying geology. Is it possible that cold-based glaciers simply have less volume of meltwater and therefore make less water available for flushing of subglacial geology? While it is true that cold-based glaciers may be smaller on average and therefore may produce less meltwater, the thermal regime of the glacier and the subglacial environment likely play more important roles. First, the lack of crevasses in cold-based systems means that meltwater is restricted to the surface and does not come into contact with geology until it reaches the proglacial area, where it flows on the surface of the moraine at atmospheric pressure. Second, the frozen ice-bed interface of cold-based glaciers means that

hydraulic conductivity is very low (see: *Irvine-Fynn et al., 2011; King et al., 2008; Kulessa and Murray, 2003;* as well as the textbooks referenced by Reviewer #3: *Bennett & Glasser, 2009; Benn & Evans, 2010; Caffey & Paterson*) and thus prevents the interaction of meltwater with subglacial geology.

Conversely, does temperate ice indicates more flushing of geologic CH4 from beneath glaciers simply because there is a larger volume of meltwater in temperate compared to cold-based glaciers? Temperate ice facilitates the interaction of meltwater and subglacial geology in several ways: (1) Temperate ice is more dynamic, which leads to crevasse formation, which provide conduits for surface meltwater to reach the glacier bed. (2) A thawed glacier bed is hydraulically conductive, allowing a subglacial drainage system to be established, which interacts with the subglacial geology.

We have added more references to the third paragraph of the introduction text where we discuss the difference between cold and temperate ice.

(2) The original manuscript's Figure 5 attempted to depict the mechanism of meltwater flushing of subglacial geological CH4. The authors removed this figure; however, such a figure is needed to more clearly communicate how temperate vs. cold ice is thought to regulate meltwater flow pathways in relation to organic-rich shales beneath glaciers.

We have added the figure back into the manuscript (which is now Figure 6), and have made some adjustments to clarify the temperate vs. cold ice zones and how their hydraulic conductivity controls the introduction of methane to the drainage system.

(3) "Frozen to the bed" indicates to me that the near-surface sediments may be frozen, but it doesn't give any information about how deeply the sediments are frozen or what their hydrologic conductivity through preferential flow paths/taliks may be. If there is permafrost beneath cold-based glaciers it seems probable that glacial meltwater in a cold-based glacier is forced to flow more deeply underground and therefore has a higher likelihood of coming into contact with CH4-rich shales. Could the authors please add subglacial permafrost to the original manuscript's Figure 5, if they think frozen beds/permafrost plays a role in the system?

We agree that subglacial groundwaters can flow deeper underground, beneath the permafrost of cold ice zones. We cannot comment on the depth of the permafrost beneath the cold ice zones (this is dependent on many factors and beyond the scope of our available data), however we can confidently state that the ice-bed interface is frozen and therefore hydrologically inactive, which prevents methane from entering the drainage system. Groundwaters flowing beneath the permafrost can acquire methane, however, these will not emerge in cold ice zones – they will likely emerge in the proglacial area. It is also important to note that cold-based glaciers do not have subglacial meltwaters because they do not produce basal meltwater since they are frozen at their beds, and their lack of crevasses means that meltwater does not penetrate to the glacier bed, but rather flows on the glacier surface.

We have indicated in Figure 6 where near-surface permafrost is likely to exist, which is beneath the cold ice zone, and throughout the glacier forefield.

(4) Perhaps this CH4-rich glacial meltwater beneath cold-based glaciers doesn't surface at the portal of the glacier (glacial terminus), but it would surface further downstream, following hydrologic gradients (and supported by downstream data in this manuscript and this team's previous work, Kleber et al. 2023). Therefore, emissions from the full length of the meltwater river systems (normalized to shale abundance and meltwater volume) may be the same for

cold-based and temperate ice glaciers. The implication may be that thermal regime doesn't impact total meltwater CH4 emissions.
To rule out this alternate possibility, the authors could calculate cumulative CH4 mass for the full length of the river normalized by shale abundance in the underlying geology and total glacial meltwater volume (i.e. total downstream river discharge) to see if there is a significant difference based on temperate ice fraction.

We appreciate the reviewer's idea but would like to reiterate here that temperate and cold glaciers will not have the same amount of meltwater infiltrating the glacier bed due to the reasons stated in the points above. Furthermore, we have found in our other work (Kleber et al., 2024) that the groundwaters emerging in glacier forefields are not necessarily of glacial origin – there are other non-glacial aquifers that can feed these, which may be the case in many cold-based systems.

While it would be interesting to determine the total methane flux from the full length of each river system, this is quite challenging and not possible with the data at hand. We know that many factors influence the change in concentration of methane observed between two points in the river: (1) outgassing, (2) methanotrophy, (3) methanogenesis, (4) inputs of methane-rich groundwaters and (5) inputs of other streams with unknown methane concentrations. Therefore, we cannot calculate a flux by assuming that all methane lost between two points was outgassed to the atmosphere. Additionally, we can also not assume that all methane introduced to the system is captured by our measurements, since a groundwater source could enter the river and partially outgas before we detect it in our measurements.

Furthermore, one of the important aspects of our findings in this manuscript, is that the groundwaters emerging throughout the glacier forefields are a more substantial methane source than the melt rivers emerging at the glacier terminus. This is important because most studies quantifying methane fluxes from glacier melt waters use samples as close to the glacier terminus as possible, meaning that they may miss a more important source of methane downstream.

To consider the reviewer's point, we have used our river flux calculations from this manuscript (calculated from the glacier termini) and the groundwater fluxes calculated from our previous work (Kleber et al., 2023) to estimate a total methane flux from subglacial river and groundwater from each system (see table below). (We were not able to do this for 4 of the glaciers because they were not included in the 2023 groundwater work, so we do not have groundwater fluxes for those.) We see that the methane mass normalized to meltwater volume does not yield values that are on a similar order of magnitude for each geological category.

**Glacier**	**Geology Category**	**Basal Temperate Ice Fraction**	**Total yearly runoff (10^6 m^3/yr)**	**Total yearl CH4 flux (kg/yr)** *Groundwater (Kleber et al, 2023) + River (this manuscript)*	**Normalized CH4 (kg CH4 / 10^6 m^3 runoff)**
Scott Turnerbreen	*1*	0.05	11.8	7	0.60
Bessemerbreen	*1*	0.00	3.2	1	0.34
Nobelbreen	*1*	0.26	19.9	15	0.76
Hettebreen	*1*	0.00	4.5	2	0.55
Svalbreen	*1*	0.10	95.6	64071	670.08

Gløttfjellbreen	*2*	0.16	16.4	760	46.40
Vallåkrabreen	*2*	0.33	79.5	22525	283.18
Mettebreen	*2*	0.34	13.0	67	5.16
Ragna Mariebreen	*2*	0.70	21.6	623	28.77
Bakaninbreen	*2*	1.00	118.0	2375	20.12
Austre Grønfjordbreen	*3*	0.42	40.8	1796	44.04
Vestre Grønfjordbreen	*4*	0.95	67.8	2562	37.79
Aldegondabreen	*4*	0.39	25.5	4	0.15
Sagabreen	*4*	0.00	5.2	2	0.44
Von Postbreen	*5*	1.00	217.9	278	1.28

If the alternate scenarios is true (that thermal regime of glacial beds does not impact total emissions from the catchment/full river system), then the major conclusion of this article should be modified to[Line 240]"Our findings indicate that the bed must be thawed and hydrologically active where the glacier meets the shale-rich geology in order for the meltwaters to effectively acquire methane by the time they reach the glacier terminus. Otherwise, for cold-based glaciers, glacial meltwater coming into contact with shale-rich geology, discharges further downstream, but is also ultimately emitted to the atmosphere."

We have shown above that the suggested alternate scenario is not true.

Lines 292-299 support this alternate statement.

We have updated these lines to highlight that this is an important finding because most glacial methane studies do not look for sources downstream when calculating fluxes and therefore important sources could be overlooked: "*Notably, peak methane concentrations occur far downstream from the glacier terminus in 68% of the rivers, indicating that the methane flux from groundwaters is potentially more critical than the methane delivered by the subglacial drainage system. This is particularly relevant for glacial catchments in more advanced stages of deglaciation, like Gløttfjellbreen, where recently exposed forefields likely contain discontinuous permafrost, allowing for intra-permafrost groundwater flow to sustain methane transport[2]. This finding highlights an important consideration for studies of methane release by glacial systems, which typically concentrate only on water emerging at the glacier terminus and may overlook important methane sources downstream.*"

We've also updated lines 176-180 to clarify that the groundwater is not necessarily of glacial origin: *"Abrupt increases in methane concentration downstream from the glacier terminus, such as those observed in the Gløttfjellebreen (2) and Vestre Grønfjordbreen (5) rivers (Fig. 3), are likely caused by discrete inputs of methane-rich groundwaters, which are commonly found in glacier forefields and can be from glacial or non-glacial sources[2,17,40–42]."*

We've also added a section on glacier surging enhancing methane mobilization, which we believe further supports our evidence that methane mobilization requires hydrologically active zones, which are increased by active surge events: "*We also find evidence that glacier surge*

events, which are becoming more frequent on Svalbard[52], may increase subglacial methane mobilization. Three of our rivers with the highest methane concentrations were actively surging or had recently finished a surge at the time of sampling (Penckbreen (11): 825 nM, Bakaninbreen (19): 857 nM, Vallåkrabreen (16): 477 nM). Sapper et al. suggested that subglacial methane production could be enhanced by glacier surging in Yukon, Canada due to the remobilization of sediments supplying fresh organic material[17]. Here, we suggest that increased basal water pressures and expansion of the hydrologically active zone due to glacier surging[53,54] enhances the flushing of subglacial methane stores."

The implications of this study may also need to be modified: Future warming should result in accelerated glacial melt with amplified flushing of CH4 from beneath glaciers AT THE GLACIER TERMINUS when the temperate ice fraction increases, but this increase AT THE GLACIER TERMINUS is likely temporary since transition towards cold-based systems will eventually reduce the hydrologic connection of meltwater to the glacial terminus stream. However, the downstream hydrology and CH4 emissions implications may need to be further assessed. How much does the dynamic at the glacial terminus impact total geologic emissions (driven by glacial meltwater traveling through groundwater) to the atmosphere along the full length of the river?

We have updated the conclusions to clarify that we are referring to methane fluxes from the subglacial drainage network, which emerges at the terminus: *"However, as glaciers thin and transition to cold-based systems, methane fluxes from subglacial drainage systems are expected to decline over time."*

Also, it is not clear from the text (line 300, section Potential emissions of methane from glacial rivers) if such a full-river analysis has been done, or if the calculations are only for the CH4 concentrations and discharge at the portal/glacier terminus. Based on Supplementary Table 1, it seems like the analysis refers to the glacial terminus only. The paper would be more meaningful in the context of glacial-CH4-climate feedbacks if a full-meltwater river analysis were conducted.

We have based our calculations only on the concentrations measured at the glacier termini. This was for several reasons: (1) This is the typical method for most glacial-methane studies, so it allows our findings to be comparable to other studies, and also allows us to point out potential shortcomings of these methods. (2) We were aiming to understand the factors controlling subglacial mobilization of methane by the subglacial drainage network, so we focused on the waters emerging at the glacier termini. (3) As discussed in an earlier point, it is not realistic to calculate a total methane flux based on the river transects we made.

We have made it more clear in the text that we are using the upstream most samples for the calculation: "We use our findings to provide a rough estimate of the amount of methane exported annually from beneath each glacier by assessing the flux of methane emerging with the melt river at each glacier terminus. By combining the methane concentrations at the glacier terminus of each river with modelled total annual runoff for each glacier[53], we estimate that…"

In lines 56-57, along with the phrase, "Cold-based glaciers have frozen beds, limiting hydraulic conductivity", please describe the conditions of cold beds in a more quantitative manner. If permafrost sediments are a characteristic of cold beds, please provide the range of depths in which the permanently frozen sediments are known or thought to occur (i.e. permafrost thickness) beneath the Svalbard glaciers, and please add the appropriate references to the manuscript.

Quantifications for the depth of permafrost beneath glaciers is very limited. On Svalbard, one study exists that shows that at depths between 35-235 m beneath glacier covered terrain, mean annual temperatures range between -1.2 and -4.9 degrees C (Christiansen et al., 2005). We do not find it appropriate to add these values to the manuscript since they are single measurements at a single site. We have rather stated that the permafrost can penetrate deep into the bedrock.

We have added the above-mentioned reference to the manuscript for lines 56-57 and updated the text: "Cold-based glaciers have frozen beds and underlying permafrost that can penetrate deep into the bedrock, which limits hydraulic conductivity[11–14]. Conversely, warm-based glaciers maintain thawed, hydrologically active beds[12–15]."

In line 58, please provide the range of depths that permanently frozen sediments can occur beneath polythermal glaciers, along with citations. If this information is not known for Svalbard, then mentioning the need for observations and modeling of subglacial permafrost hydrology as a way to improve understanding of glacial meltwater CH_4 dynamics would be appropriate in the Discussion. Since strong CH_4 emissions are known to occur on Svalbard through intrapermafrost groundwater flow pathways (Hodson 2020), such flowpaths also likely occur beneath glaciers. This implies that glacial meltwater in cold-based glaciers likely finds a way to flow down through frozen sediments and come into contact with shale-rich horizons. Discharge to the river at the portal may not occur under the cold-based glaciers, but likely does occur further downstream. It seems that previous work by these authors (Kleber et al. 2023) supports this concept.

Please see the above point with the included citation.

Summary:
-Figure 5 from the previous manuscript should be returned to the paper so that it depicts the authors' interpretation of subglacial hydrologic connections between meltwater and underlying geology for temperate vs. cold ice. Please include permafrost if/where it is thought to occur, and how these frozen sediments may regulate subglacial hydrologic flow pathways.

Please see points 2 and 3.

-The paper would be more meaningful in the context of glacial-CH_4-climate feedbacks if a full-meltwater river analysis were conducted.

Please see point 4.

- Given that the novelty of glacier meltwater flushing geologic CH_4 in this region has already been published by these authors (Kleber et al. 2023), and that this paper is trying to work out the relationship between temperate vs. cold ice in controlling hydrologic connection to the underlying geology, I wonder if this paper would be better suited for a more specialized journal.

We agree with Reviewer #3 that this study is the first of its kind and represents an extremely valuable dataset that offers the first upscaled estimation of methane release from subglacial meltwaters on Svalbard. Our findings are important globally for constraining and predicting carbon inventories and fluxes.

Other comments:
Line 75. Please support the statement that CH_4-producing soils and wetlands are not expansive

and therefore likely a smaller source than glacial catchments by providing quantitative values. The authors' statement may reflect truth; however, supporting information is needed since hotspots occupy small areas but can dominate emissions. For instance, it would be useful to present the details (area x CH4 flux) for both wetlands and glacial catchments on the archipelago. If all of this information is not available (e.g. because the glacial flux estimate is the goal of this study), then please change the language "are likely' to "may be" in line 77; but regardless, please provide the areas of each land cover type (organic rich soils/wetlands vs. glacial catchments) and whatever fluxes are already known.

We have updated the text with the available numbers as suggested: *"Given that glaciers cover nearly 60% of the archipelago[22] and methane-rich source rocks occupy almost half of Svalbard's land surface area[23], the methane emissions from this process are expected to exceed those from permafrost thaw in the region[18,24]. While permafrost thaw on Svalbard can result in high methane emissions per unit area[25], the organic-rich soils and wetlands that yield high rates of methane production are not expansive on the archipelago. Consequently, methane emissions from Svalbard's extensive glacial catchments may be a more significant source of methane to the atmosphere[24]."*

The upscaled estimates of glacial meltwater emissions from this study are provided in line 320. Please provide the estimate for organic-rich soil/wetland emissions as a comparison, along with what is reported for groundwater emissions in lines 339-349.

Unfortunately, there are no upscaled estimates for methane flux from permafrost thaw on Svalbard. The emission estimate from groundwaters is already included in these lines (2.31 kt CH4/yr).

We have removed the comparison to permafrost emissions in this discussion, as we acknowledge that it is perhaps too speculative.

Line 99. The isotopic composition alone does not indicate CH4 origin. Please change to "the isotopic composition of methane combined with the wetness ratio (C1/(C2+C3)) indicates the likely origin, thermogenic and/or microbial sources."

We have updated the lines as suggested.

Line 105. The abstract says "425 times"; this line says "400 times". These values are close, but should probably be consistent in the text.

Thank you for catching this! We have updated the line to say 425 times.

Supplementary Table 1: "bRunoff from ref. XXX (Louise's model)". This reference still needs to be corrected. Please see Reviewer 2 comments from the previous round.

Thank you for catching this! We failed to update the supplementary with the updated table in the last review. It is now fixed.

Reviewer #3 (Remarks to the Author):

This is an field-based study that investigates whether proglacial rivers across Svalbard are a significant source of methane and the factors that control the subglacial methane release. It focuses on 19 glacier catchments where 148 water samples were collected and analysed for methane concentration and carbon isotopic composition to identify methane fluxes and their specific sources. The paper also uses radar - GPR to characterize subglacial thermal regimes and the extent of thawed versus frozen glacier substrates, and is used this to determine how methane concentrations vary with underlying geology, glacier size, and hydrological conditions.

Results reveal that methane supersaturation is widespread in Svalbard's glacial meltwaters, with concentrations attaining up to 400 times atmospheric equilibrium levels. Isotopic and hydrocarbon analyses demonstrate that most of the methane is thermogenic rather than biogenic origin, derived from organic-rich geological formations of shale and coal-bearing rocks rather than primarily from microbial production. The highest methane concentrations occur where glaciers overlie shale-rich bedrock and possessed temperate (i.e. thawed), hydrologically active beds that facilitate active methane transport by subglacial discharge. Although microbial methane production and oxidation occurred in some rivers, these processes were secondary to the dominant geological source.
The authors conclude that glacier meltwaters represent an important pathway for mobilizing ancient (thermogenic) methane stores and that continued glacier retreat will initially enhance methane release through enhanced subglacial hydrological activity. Particularly, the authors speculate that such hydrological conditions might drive substrate hydrofracture, promoting further geological methane flow from shales. However, emissions may eventually decline as glaciers transition to cold-based conditions that limit subglacial meltwater flow and methane transport. The findings highlight a potentially critical climate feedback mechanism and provide a framework for assessing methane emissions from glacierized catchments worldwide.

As far as I am aware of the (considerable) literature on Arctic Svalbard, it is a first of its kind and in my view - will become a benchmark "go to" study. The authors have undertaken a huge amount of work in terms of the field effort to collect the samples, GPR data etc, carried out the hydrocarbon and isotopic analyses, collated these data against the geological controls to arrive at substantive and robust conclusions as to the origin and controls of the CH4 release across Svalbard. The paper is very well written and presents robust reasoning, justification and arguments. The results and conclusions are important not just locally, but globally for constraining carbon inventories and fluxes. The wider discussion of controls on CH4 origin and release including glacier thermal regime, subglacial hydrology, geology/lithology is logical and really very well honed. The paper is nicely executed and appropriately pitched for the journal and I foresee that it will be very well cited and, as said, a benchmark for subglacial methane release from this important Arctic region.

I recommend it for publication in Nature Communications with just one minor comment. It has already been iterated through the review process and I would judge that it has benefited in

clarity, focus and polish from previous revision(s).

My only comment is that I would like to see a wider and deeper discussion of the methane fluxes (to the atmosphere) here compared with those that have recently been published from rivers emerging along the entire flank of the western Greenland ice sheet (Hatton et al., 2026). The origin of methane from this recently published Greenland study is biogenic and (as pointed out) quite different from the predominantly thermogenic sources identified in this study. Still, it would be useful if the authors could present and evaluate the actual methane fluxes and their relative importance from these contrasting geology/settings and the implication for future subglacial sourced methane release from polar ice sheets given their respective geological settings. Specifically, are the results from this Svalbard (hydrocarbon rich / geothermal) setting significant in terms of the potential geology underlying parts of Greenland and the Antarctic ice sheets where significant hydrocarbon-rich provinces have been mapped/inferred?

We thank Reviewer #3 for taking the time to provide their constructive comments and suggestions. We agree that a discussion around the contrasting methane sources between Greenland and Svalbard is warranted and highly valuable in this manuscript.

We have added a paragraph (lines 391-403) to address this: "*Our upscaled Svalbard-wide methane flux estimate is a similar order of magnitude to the lower-end estimate for methane emissions from glacial rivers across Greenland (918-2110 t CH_4 yr^{-1})[61]. This is substantial given that Greenland ice cover is more than 55-times larger than that of Svalbard. This disparity reflects the contrasting origins of methane between the two regions. Subglacial methane beneath Greenland's glaciers is of microbial origin, produced in situ from organic material overridden during past glacial advances[61]. In contrast, Svalbard's shale and carbon-rich geology supersaturates subglacial meltwaters with ancient thermogenic methane, which overwhelms any potential microbial source. This geologic source is evidently more effective at supersaturating meltwaters and thus yields disproportionately higher methane fluxes. These results implicate other glaciated regions that are situated within significant hydrocarbon-rich geological provinces, such as parts of Arctic Russia, Canada, northeast Greenland and sections of Antarctica[5,62], where glacial methane emissions have yet to be investigated."*

Finally, I did briefly look at the author's rebuttal to previous reviews, and feel it is appropriate to comment on what appears to be a misunderstanding from Reviewer 1 regarding the polythermal characterisation (i.e. mixed temperate and cold bedded nature) of Svalbard's glaciers. There is a considerable glaciological literature establishing that many of Svalbard's glaciers are cold-bedded, specifically as they thin and retreat and fail to insulate from a very cold atmospheric signal (see for example, textbooks: Bennett & Glasser, 2009; Benn & Evans, 2010; Caffey & Paterson supplemented by wide-ranging reviews by Irvine-Fynn et al., 2011; etc). My understanding is that the classification "polythermal" glacier was first defined in relation to Svalbard, and it is well established that the ice fabric and beds of glaciers are mixed cold and temperate ice. For this reason, I found Reviewer #1's preoccupation that Svalbard glaciers are temperate bedded quite misled and in my view, the authors are correct to rebut this

misconception quite robustly.

References:
Benn, D. and Evans, D. 2010 Glaciers and Glaciation. Second Edition. London, Routledge. https://doi.org/10.4324/9780203785010

Bennett, M. and Glasser, N.F. 2009. Glacial geology: ice sheets and landforms. Second edition. Oxford, Wiley-Blackwell.

Cuffey, K. and Paterson, W.S.B. 2010. The physics of glaciers. Fourth edition. Amsterdam, etc., Academic Press. ISBN-10: 0-123694-61-2.

Hatton, J.E. et al. 2026. Mid-Holocene retreat of the Greenland Ice Sheet indicated by subglacial methane release. Nature Geoscience, pp.1-7.

Irvine-Fynn, T.D. et al., 2011. Polythermal glacier hydrology: A review. Reviews of Geophysics, 49(4).

Reviewer #1 (Remarks to the Author):

This round of revisions and responses to the author has cleared up my misunderstanding and concerns. The critical point that has been resolved is that meltwater for cold-based glaciers runs off at the surface and does not infiltrate into bedrock beneath these glaciers. With this clarification, the interpretation and conclusions make sense and are both interesting and significant. Could the authors add arrows to show this surface runoff for the cold-ice (cold-based portion) of the glacier in Fig. 6? For a broad audience, this would be helpful. I agree with Reviewer 3 that the investigation represents a tremendous amount of work and will be a keystone paper. The comparisons of the thermogenic-CH4 fluxes in Svalbard to those of microbially-dominated CH4 in Greenland makes a strong addition to the revised manuscript.

We thank the reviewer for their thorough and constructive review of our manuscript. We have added arrows in Figure 6 to show the surface runoff for the cold-ice portion of the glacier.

Reviewer #3 (Remarks to the Author):

I thank the authors for submitting a revised manuscript that adds a paragraph comparing their upscaled (predominantly thermogenic-source) methane results from across Svalbard with up-to-date estimates of that released (biogenic) from the Greenland ice sheet. This small section is neatly written, honed and a useful dimension to the discussion, better placing it within an Arctic - and global - context.

In terms of Reviewer#1's comments - specifically as to whether surface meltwater can access the basal interface of cold / frozen glaciers, the author's have carried out an authoritative and comprehensive rebuttal to this critique. The literature - including major glaciology text books (which they cite) as well as major reviews of polythermal glacier hydrology (also now cited), demonstrate that hydrological systems associated with temperate and cold bedded glaciers are markedly different. The authors' description of cold based glacier regimes - and how it impacts the drainage system - limiting supraglacial meltwater access to the basal environment is quite correct and in-line with all the known literature. Furthermore, their inferences in terms of methane mobilization under contrasting temperate and cold basal conditions are also sound.

My evaluation is that the authors are robust in their analysis and expansive assessment of the mechanisms driving subglacial methane mobilization and release across Svalbard. Given the large inventory of glaciers monitored, along with the excellent geophysical datasets analyzed to support their analysis, this study represents a major contribution to the literature, and will become a "go to" case reference that will no doubt be highly cited over time.

I recommend publication in Nature Communications without further ado.

We thank the reviewer for their constructive suggestions for our manuscript.
