# Supplementary material for: Subglacial geology and thermal conditions regulate methane emissions from Svalbard glaciers
Source: Nat Commun. 2026 Sep 8;17:9347. doi: 10.1038/s41467-026-77190-z (PMC13554054; doi:10.1038/s41467-026-77190-z)
Supplement: Supplementary file 3 [file 41467_2026_77190_MOESM3_ESM.pdf]

Description of Additional Supplementary Files

File Name: Supplementary Data 1
Description: **Summary of transect sampling measurements**
Includes measured hydrochemical data from each sampling point in this study (methane concentrations, carbon isotopic composition of methane, wetness, electrical conductivity, dissolved oxygen and pH)
